# Matrix metalloproteinases and their tissue inhibitors as upcoming biomarker signatures of connective tissue diseases-related interstitial lung disease: towards an earlier and accurate diagnosis

**DOI:** 10.1186/s10020-025-01128-2

**Published:** 2025-02-20

**Authors:** Verónica Pulito-Cueto, Belén Atienza-Mateo, Joao C. Batista-Liz, María Sebastián Mora-Gil, Víctor M. Mora-Cuesta, David Iturbe-Fernández, Sheila Izquierdo Cuervo, Carolina Aguirre Portilla, Ricardo Blanco, Raquel López-Mejías

**Affiliations:** 1 Immunopathology Group, Marqués de Valdecilla University Hospital-IDIVAL, Santander, Spain; 2 Department of Rheumatology, Hospital Universitario Marqués de Valdecilla, Santander, Spain; 3 Department of Pneumology, Hospital Universitario Marqués de Valdecilla, Santander, Spain

**Keywords:** Matrix metalloproteinases, Matrix metalloproteinases tissue inhibitors, Interstitial lung disease, Autoimmune diseases, Rheumatoid arthritis, Systemic sclerosis, Biomarkers, Pulmonary fibrosis

## Abstract

**Background:**

Lack of understanding of interstitial lung disease (ILD) associated with systemic sclerosis (SSc) and rheumatoid arthritis (RA) hinders the early and accurate identification of these devastating diseases. Current clinical tools limitations highlight the need to complement them with accessible and non-invasive methods. Accordingly, we focused on identifying useful matrix metalloproteinases (MMPs) and their tissue inhibitors (TIMPs) as new biomarkers with clinical value in the diagnosis and prognosis of RA-ILD^+^ and SSc-ILD^+^.

**Methods:**

Peripheral blood was collected from patients with RA-ILD^+^ (*n* = 49) and SSc-ILD^+^ (*n* = 38); as well as with RA-ILD^-^ (*n* = 25), SSc-ILD^-^ (*n* = 20) and idiopathic pulmonary fibrosis (IPF) (*n* = 39). MMP-1, MMP-2, MMP-3, MMP-7, MMP-9, MMP-10, MMP-12, TIMP-1, and TIMP-2 serum levels were measured using xMAP Technology.

**Results:**

Concerning early connective tissue disease (CTD)-ILD^+^ diagnosis, increased MMP-7, MMP-9, MMP-10, and MMP-12 levels were found in RA-ILD^+^ and SSc-ILD^+^ patients in relation to RA-ILD^-^ and SSc-ILD^-^ patients, respectively. RA-ILD^+^ patients showed higher MMP-2 levels and lower TIMP-1 levels than RA-ILD^-^ patients. Interestingly, a reliable utility for identifying ILD in CTD was confirmed for the MMP-2, MMP-7, MMP-9, MMP-10, MMP-12, and TIMP-1 combination in RA and MMP-7, MMP-9, MMP-10, and MMP-12 combinatorial signature in SSc. Regarding accurate CTD-ILD^+^ diagnosis, RA-ILD^+^ and SSc-ILD^+^ patients showed lower MMP-7 and MMP-10 levels than IPF patients. Lower MMP-9 and TIMP-1 levels and higher MMP-3 levels were found in RA-ILD^+^ compared to IPF. Remarkably, effectively better differentiation between CTD-ILD^+^ and IPF was confirmed for a 5-biomarker signature consisting of MMP-3, MMP-7, MMP-9, MMP-10, and TIMP-1 in RA as well as for the MMP-7 and MMP-10 combination in SSc. Finally, in RA-ILD^+^ patients, higher MMP-10 levels were associated with worse pulmonary function, increased MMP-2 levels were related to the treatment with conventional synthetic disease-modifying anti-rheumatic drugs, and decreased TIMP-1 levels were linked with positivity rheumatoid factor status.

**Conclusions:**

MMPs and TIMPs form combinatorial biomarker signatures with clinical value for non-invasive, early, and accurate diagnosis of RA-ILD^+^ and SSc-ILD^+^, constituting promising screening tools in clinical practice.

**Supplementary Information:**

The online version contains supplementary material available at 10.1186/s10020-025-01128-2.

## Introduction

Interstitial lung disease (ILD) is one of the most common and potentially fatal complications in connective tissue disease (CTD) patients, with rheumatoid arthritis (RA) and systemic sclerosis (SSc) being the most affected, accounting for approximately 39% and 31% of these CTDs-ILD, respectively (Guiot et al. 2024; Cerro Chiang and Parimon 2023). The onset of CTD-ILD is insidious, and the clinical symptoms are atypical and not obvious, which may explain why ILD is often diagnosed in later stages with severe consequences (Guiot et al. 2024; Matson and Demoruelle 2024). Assessments of signs and symptoms, pulmonary function tests (PFTs), and high-resolution computed tomography (HRCT) are currently used to diagnose and monitor CTD-ILD patients (Guiot et al. 2024). PFTs are very useful for monitoring the progression of ILD, but they are not specific enough to be used as diagnostic tools since lung function declines slowly and is not easy to detect (Guiot et al. 2024). In fact, patients in earlier phases of ILD may be asymptomatic and still have normal lung function (Guiot et al. 2024). Thus, HRCT is the gold standard in the diagnosis of pulmonary fibrosis, but it has a certain delay, there is a low to moderate agreement among expert radiologists interpreting it, and it is associated with radiation harmful to humans, which means it requires careful indication (Guiot et al. 2024). Consequently, the early diagnosis of ILD in patients with CTD remains often a challenge for clinicians and, given its poor prognosis and emerging immunomodulatory and antifibrotic treatment options (Matson and Demoruelle 2024), there is considerable interest in addressing this problem. Additionally, a confident diagnosis of CTD-ILD can be complicated due to certain similarities with other ILDs, particularly with idiopathic pulmonary fibrosis (IPF), the most severe and prevalent ILD (Enomoto 2024). The prognosis and therapies of CTD-ILD are very different from other ILDs, and adequate diagnosis and treatment are crucial to delay the progression of fibrosis (Enomoto 2024). However, how to predict and diagnose this group of patients remains difficult and unclear (Enomoto 2024).

The often-unrecognized CTD-ILD and the associated morbidity/mortality highlight the need for clinical tools for its early and accurate diagnosis (Cerro Chiang and Parimon 2023). Given the clinical implications of the signaling cascades involved in these processes, circulating biomarker levels are promising candidates to complement the drawbacks of HRCT and PFTs, representing attractive tools as an accessible and less invasive diagnostic method that can be used to identify factors governing disease pathogenesis and progression. Demonstrating the feasibility of this approach, our group and other authors have recently found serum biomarkers to be of clinical value in the diagnosis of ILD, being broadly studied in the most common ILD, IPF (Wang et al. 2023a; Zhu et al. 2023) and, to a lesser extent, in CTD-ILD (Cerro Chiang and Parimon 2023; Ahmed and Handa 2022; Stainer et al. 2023; Hoffmann-Vold et al. 2020; Pulito-Cueto et al. 2022, 2023a, b). However, the use of these biomarkers has yet to gain widespread clinical use due to insufficient research evidence, thus significantly increased research efforts are required in this field.

In this context, the primary feature of lung fibrosis is the massive deposition of extracellular matrix (ECM) by myofibroblast, which causes the enlargement of the interstitial space, increasing the distance between circulating blood and alveoli, replacing air content with ECM (Cerro Chiang and Parimon 2023; Wang et al. 2024; Chuliá-Peris et al. 2022). This pathological process eventually leads to impaired diffusion and ventilation and respiratory failure (Cerro Chiang and Parimon 2023; Wang et al. 2024; Chuliá-Peris et al. 2022). Therefore, the abnormal ECM remodeling is a hallmark of ILD, and matrix metalloproteinases (MMPs) and their tissue inhibitors (TIMPs) have been proposed to be key in causing these pathologic changes as they are directly responsible for the degradation of the ECM (Atkinson and Senior 2003; Vandenbroucke et al. 2011; Klein and Bischoff 2011; Brew et al. 2000). Remarkably, it exits great evidence that links the alteration of MMPs and TIMPs levels with the pathogenesis of IPF based upon the results of studies reporting elevated levels of these proteins, including MMP-1, MMP-2, MMP-7, MMP-9, MMP-12, and TIMP-1, in blood samples of IPF patients, and highlighting MMP-7 as a diagnostic and prognostic biomarker of the disease (Wang et al. 2023a; Atkinson and Senior 2003; Balci et al. 2023; Inoue et al. 2020; Craig et al. 2015; Pardo and Selman 2006; Richards et al. 2012; Rosas et al. 2008; Song et al. 2013; Todd et al. 2020; Tzouvelekis et al. 2017). Notably, MMPs and TIMPs have emerged as promising targets for the treatment of IPF (Craig et al. 2015; Peng et al. 2022). However, until now, studies focused on ECM remodeling-related biomarkers for early and accurate diagnosis of CTD-ILD are limited and insufficient to reach their application in clinical practice(Chen et al. 2023; Lv et al. 2022).

Based on these considerations, there remains a high unmet need to specifically identify CTD-ILD patients in the early stages of the disease to prevent progression and avoid irreversible lung damage and mortality. In this sense, we hypothesize that the evaluation of ECM remodeling-related biomarker panels may complement the ability of currently used physiological and imaging parameters to diagnose CTD-ILD, thus addressing the challenge posed by this troubling disease.

Accordingly, we focused on identifying useful MMPs and TIMPs as novel biomarkers with clinical value in the diagnosis and prognosis of ILD in the most affected CTDs for this devastating disease, SSc and RA.

## Methods

### Patient populations

The population included in this study comprises two study objective groups of patients with CTD-ILD^+^: 49 patients with RA-ILD^+^ and 38 patients with SSc-ILD^+^; and three comparative patient groups: 25 with RA-ILD^-^, 20 with SSc-ILD^-^, and 39 with IPF.

RA patients met the 2010 American College of Rheumatology (ACR)/ European League Against Rheumatism (EULAR) criteria(Aletaha et al. 2010) and SSc patients fulfilled the 2013 ACR/ EULAR criteria (Hoogen et al. 2013). Pulmonary involvement was evaluated in all the patients by HRCT images of the chest and PFTs. Patients with each CTD were classified by the presence or absence of ILD according to the American Thoracic Society (ATS) / European Respiratory Society (ERS) criteria for ILD (Travis et al. 2013). IPF patients met the ATS/ERS criteria (Travis et al. 2013).

All these individuals were recruited from the Rheumatology and Pneumology department of Hospital Universitario Marqués de Valdecilla (Santander, Spain). Peripheral venous blood samples and demographic and clinical features including sex, age, smoking history, C-reactive protein, erythrocyte sedimentation rate, antibody status, PFTs, and HRCT patterns were collected from patients. In particular, HRCT patterns of ILD patients were stratified according to the criteria for the usual interstitial pneumonia pattern of the Fleischner Society (Lynch et al. 2018). In addition to HRCT patterns, ILD patients were also stratified according to the presence/absence of progressive pulmonary fibrosis following the ATS/ERS/Japanese Respiratory Society/Latin American Thoracic Society criteria (Raghu et al. 2022) (Additional File 1: Table S1).

All the experiments involving humans and human blood samples were carried out in accordance with the approved guidelines and regulations, according to the Declaration of Helsinki. All experimental protocols were approved by the Ethics Committee of Clinical Research of Cantabria, Spain (2016.092). All subjects gave written informed consent to participate in this study before their inclusion.

### MMPs and TIMPs serum level assays

MMP-1, MMP-2, MMP-3, MMP-7, MMP-9, MMP-10, MMP-12, TIMP-1, and TIMP-2 serum levels were assessed by a xMAP^®^ Technology (HMMP1MAG-55 K-03, HMMP2MAG-55 K-05, HTMP1MAG-54 K-02, Merck Millipore, Darmstadt, Germany) following the manufacturer’s instructions. Commercial and proprietary positive and negative controls were included to ensure the reliability of our results. All the samples and commercial and proprietary controls were evaluated in duplicate and analyzed in the Luminex^®^ 200™.

### Statistical analyses

Continuous and categorical variables were expressed as mean ± standard deviation (SD) and as well as number (n) and percentage (%) of individuals, respectively.

To identify potential specific biomarkers of CTD-ILD^+^, the MMP and TIMP level differences between CTD-ILD^+^ and CTD-ILD^-^ patients as well as CTD-ILD^+^ and IPF patients were evaluated by analysis of variance (ANOVA), adjusting for the following potential confounding factors: sex, age at the time of the study, and smoking history.

ROC analysis was performed when statistically significant protein variations were found between groups to evaluate their discriminative capacity. The Youden index (the highest value obtained from the formula sensitivity% + specificity% − 100) was used to calculate the optimal cut-off values of those proteins for discriminating between two groups of patients. Moreover, ROC curves were generated to identify if a combinatorial signature composed of several biomarkers improved the discriminative capacity of these proteins separately. The AUC with a 95% confidence interval (CI) was calculated for each biomarker of interest and the combination of these investigational proteins.

To evaluate the association of MMPs and TIMPs with CTD-ILD^+^ severity, the relationship of protein levels with continuous (PFTs) and categorical variables (radiological pattern and presence/absence of progressive pulmonary fibrosis) was analyzed via the estimation of Pearson’s partial correlation coefficient (r) and linear regression, respectively, adjusting for the potential confounding factors above mentioned.

To assess the relationship of MMPs and TIMPs with the CTD-ILD^+^ prognostic, Kaplan–Meier graphs were constructed using the ROC threshold of the protein levels that discriminate RA-ILD^+^ and SSc-ILD^+^ from IPF to obtain a dichotomous variable and to represent cumulative survival (time to death or lung transplantation). The log-rank test was carried out to analyze differences in RA-ILD^+^ and SSc-ILD^+^ patients in time to death or lung transplantation between those with lower and higher levels of MMPs and TIMP than the optimal cut-off values.

To assess the relationship of MMPs and TIMPs with other clinical characteristic of CTD-ILD^+^, the association of the levels of the significant proteins in ROC curves with continuous (CTD and ILD duration) and categorical (antibody status, treatment, and other SSc clinical manifestations) variables was analyzed via the estimation of Pearson’s partial correlation coefficient (r) and linear regression, respectively, adjusting for the potential confounding factors above mentioned.

Statistically significant differences were considered as *p* < 0.05. Statistical analyses were performed using STATA statistical software 12/SE (Stata Corp., College Station, TX, USA).

## Results

### Alterations of MMPs and TIMPs serum levels are associated with the presence of ILD in patients with RA and SSc

**Fig. 1:**
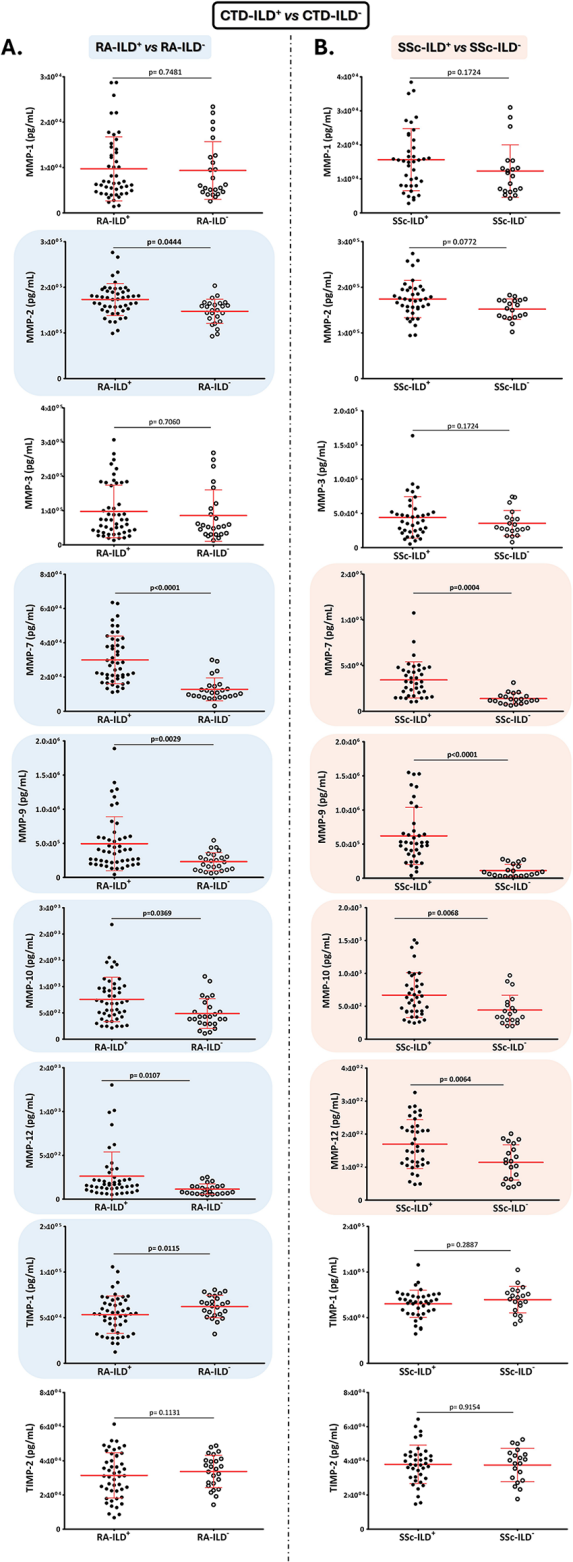
**Role of MMPs and TIMPs in the ILD diagnosis in patients with CTDs**. Differences in serum levels of MMP-1, MMP-2, MMP-3, MMP-7, MMP-9, MMP-10, MMP-12, TIMP-1, and TIMP-2 between patients with RA-ILD^+^ and RA-ILD^-^ (**A**), as well as patients with SSc-ILD^+^ and SSc-ILD^-^ (**B**). MMPs: matrix metalloproteinases; TIMPs: Matrix metalloproteinases inhibitors; ILD: interstitial lung disease; CTDs: connective tissue diseases; RA: rheumatoid arthritis; SSc: systemic sclerosis. Significant results are highlighted

Increased serum levels of MMP-7, MMP-9, MMP-10 and MMP-12 were found in patients with RA-ILD^+^ and SSc-ILD^+^ in relation to RA-ILD^-^ and SSc-ILD^-^ patients, respectively (RA: *p* < 0.0001, *p* = 0.0029, *p* = 0.0369, *p* = 0.0107, Fig. 1A; and SSc: *p* = 0.0004, *p* < 0.0001, *p* = 0.0068, *p* = 0.0064, Fig. 1B; respectively).

Specifically, patients with RA-ILD^+^ showed higher levels of MMP-2 and lower levels of TIMP-1 compared to those with RA-ILD^-^ (*p* = 0.0444 and *p* = 0.0115, respectively, Fig. 1A).

Regarding MMP-1, MMP-3, and TIMP-2, no statistical differences were observed between patients with CTD-ILD^+^ and those with CTD-ILD^-^, regardless of the underlying CTD (Fig. 1A and B).

### Changes in MMPs and TIMPs serum levels characterize patients with RA-ILD^+^ and SSc-ILD^+^*versus* IPF patients

**Fig. 2:**
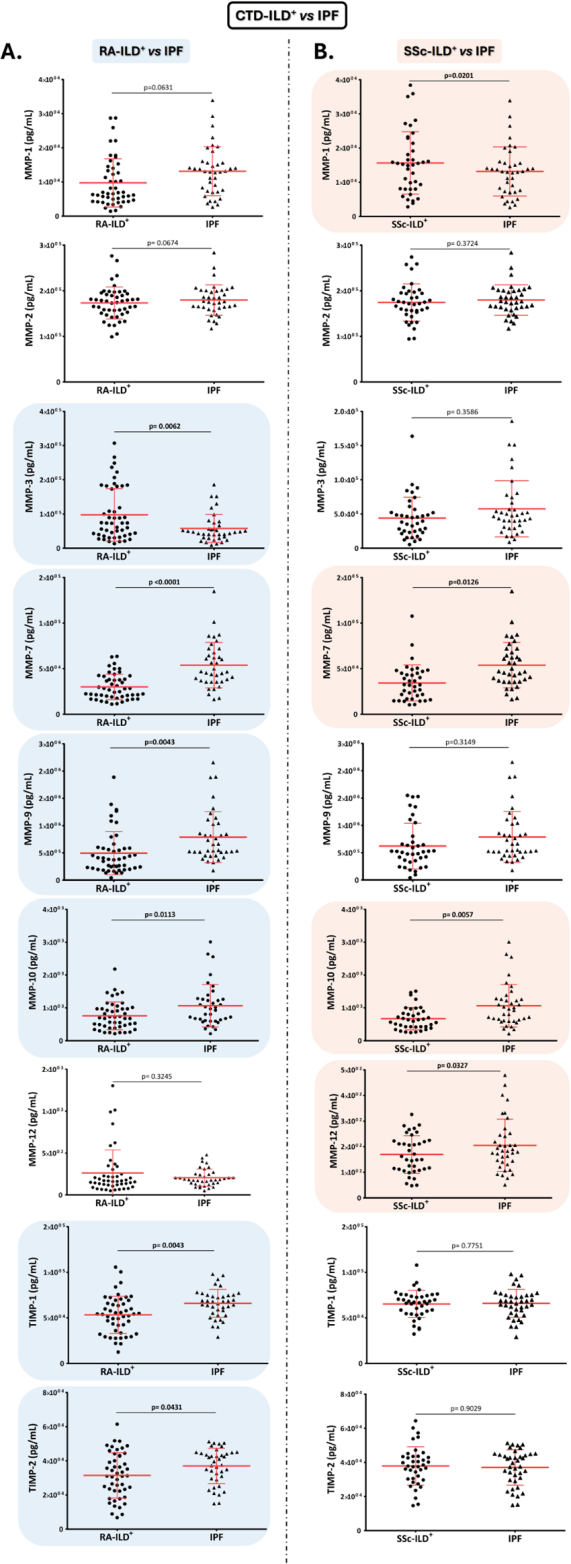
**Role of MMPs and TIMPs in the differential diagnosis between patients with CTDs-ILD**^**+**^ **and IPF**. Differences in serum levels of MMP-1, MMP-2, MMP-3, MMP-7, MMP-9, MMP-10, MMP-12, TIMP-1, and TIMP-2 between patients with RA-ILD^+^ and IPF (**A**), as well as patients with SSc-ILD^+^ and IPF (**B**). MMPs: matrix metalloproteinases; TIMPs: Matrix metalloproteinases inhibitors; ILD: interstitial lung disease; CTDs: connective tissue diseases; RA: rheumatoid arthritis; SSc: systemic sclerosis: IPF: idiopathic pulmonary fibrosis. Significant results are highlighted

Patients with RA-ILD^+^ and SSc-ILD^+^ showed lower serum levels of MMP-7 and MMP-10 than those with IPF (RA: *p* < 0.0001, *p* = 0.0113, Fig. 2A; and SSc: *p* = 0.0126, *p* = 0.0057, Fig. 2B; respectively).

Particularly in RA-ILD^+^ patients, MMP-9, TIMP-1, and TIMP-2 were also decreased compared to the levels of these molecules in patients with IPF (*p* = 0.0043, *p* = 0.0043 and *p* = 0.0431, respectively, Fig. 2A). Moreover, MMP-3 levels were higher in RA-ILD^+^ patients than those with IPF (*p* = 0.0062, Fig. 2A).

Specifically, patients with SSc-ILD^+^ showed higher levels of MMP-1 and lower levels of MMP-12 in relation to patients with IPF (*p* = 0.0201 and *p* = 0.0327, respectively, Fig. 2B).

About MMP-2, no statistical differences were observed between patients with RA-ILD^+^ and SSc-ILD^+^, and those with IPF (Fig. 2A and B).

### MMPs and TIMPs constitute combinatorial biomarker signatures for the ILD diagnosis in patients with RA and SSc

**Fig. 3:**
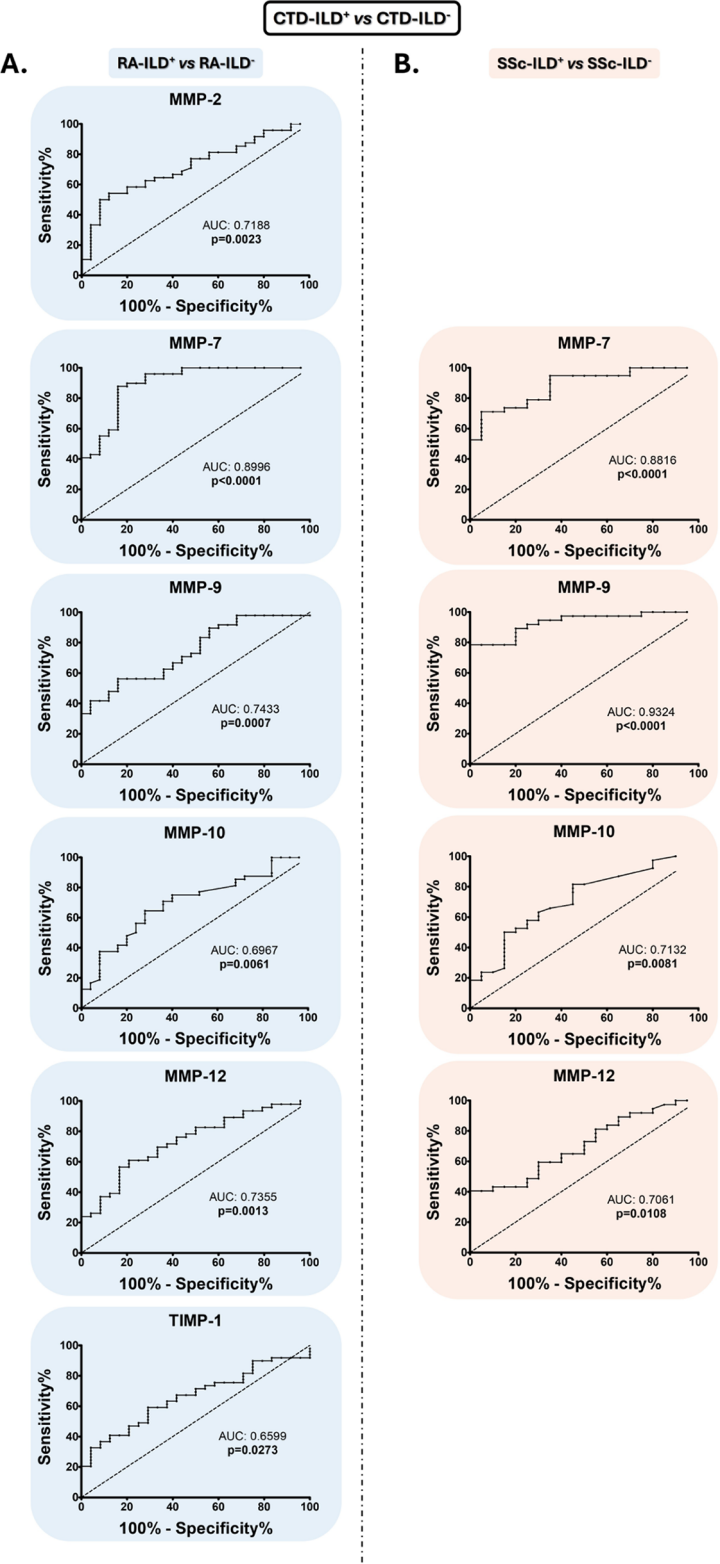
**Utility of MMPs and TIMPs as biomarkers of the ILD presence in SSc and RA patients**. ROC curve analysis of MMPs and TIMPs for the discrimination of RA-ILD^+^ from RA-ILD^−^ (**A**) and SSc-ILD^+^ from SSc-ILD^−^ (**B**). CTD: connective tissue disease; ILD: interstitial lung disease; RA: rheumatoid arthritis; SSc: systemic sclerosis; MMPs: matrix metalloproteinases; TIMPs: Matrix metalloproteinases inhibitors; AUC: area under the curve. Significant results are highlighted

**Fig. 4:**
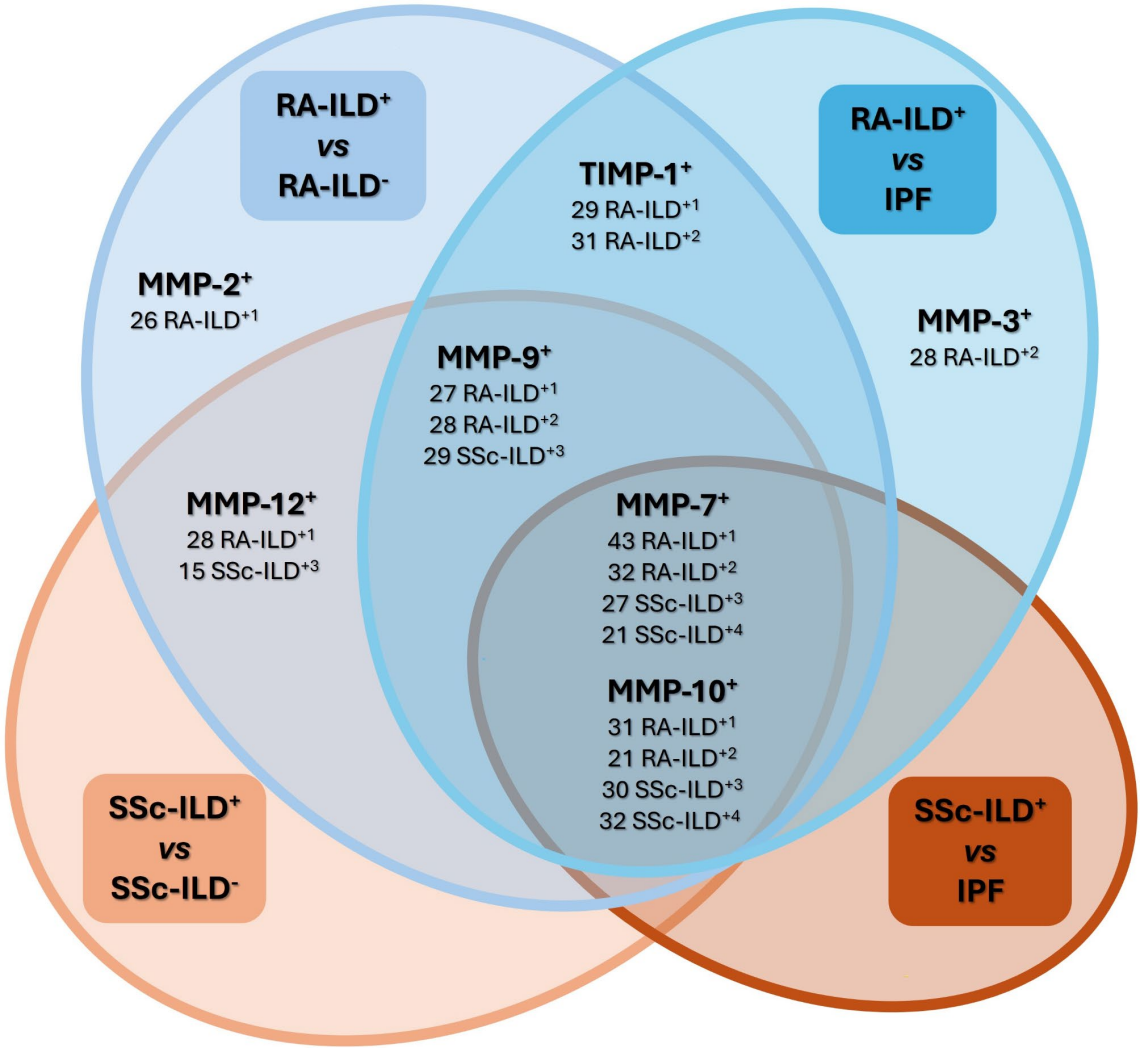
**Venn diagram represents the distribution of the positivity of each biomarker in RA-ILD**^**+**^ **and SSc-ILD**^**+**^ **patients for their characterization** ***versus*** **RA-ILD**^**−**^ **and SSc-ILD**^**−**^**, respectively, and IPF patients.** The diagram shows the number of RA-ILD^+^ and/or SSc-ILD^+^ patients who were positive for the biomarker cut-off values defined to the characterization of RA-ILD^+^*versus* RA-ILD^−^ patients^1^, RA-ILD^+^*versus* IPF patients^2^, SSc-ILD^+^*versus* SSc-ILD^−^ patients^3^, and SSc-ILD^+^*versus* IPF patients^4^. RA: rheumatoid arthritis; ILD: interstitial lung disease; IPF: idiopathic pulmonary fibrosis; SSc: systemic sclerosis; MMPs: matrix metalloproteinases; TIMPs: Matrix metalloproteinases inhibitors

Interestingly, we found that MMP-7, MMP-9, MMP-10, and MMP-12 were useful as diagnostic tools for identifying the presence of ILD in patients with RA and SSc (area under the curve (AUC): 0.8996, *p* < 0.0001, AUC: 0.7433, *p* = 0.0007, AUC: 0.6967, *p* = 0.0061, AUC: 0.7355, *p* = 0.0013, respectively, Fig. 3A, for RA-ILD^+^; and AUC: 0.8816, *p* < 0.0001, AUC: 0.9324, *p* < 0.0001, AUC: 0.7132, *p* = 0.0081, AUC: 0.7061, *p* = 0.0108, respectively, Fig. 3B, for SSc-ILD^+^, Additional File 2: Table S2). The optimal cut-off value for MMP-7, MMP-9, MMP-10, and MMP-12 that achieved the best sensitivity and specificity were > 15,806 pg/mL, > 347,110 pg/mL, > 523.8 pg/mL and > 146.8 pg/mL, respectively, for the detection of ILD in RA patients (Additional File 2: Table S2), as well as > 21,564 pg/mL, > 302,893 pg/mL, > 382.1 pg/mL, > 203.7 pg/mL, respectively, for the identification of ILD in SSc patients (Additional File 2: Table S2). In particular, the number of patients who were positive for these defined cut-off values was, in the case of RA-ILD^+^ patients, 43 for MMP-7, 27 for MMP-9, 31 for MMP-10, and 28 for MMP-12, and regarding SSc-ILD^+^ patients, 27 for MMP-7, 29 for MMP-9, 30 for MMP-10 and 15 for MMP-12 (Fig. 4).

Furthermore, the ability of MMP-2 and TIMP-1 serum levels to characterize RA-ILD^+^*versus* RA-ILD^-^ was confirmed by ROC analyses (AUC: 0.7188, *p* = 0.0023 and AUC: 0.6599, *p* = 0.0273, respectively, Fig. 3A, Additional File 2: Table S2) determining > 169,948 pg/mL for MMP-2 and < 56,004 pg/mL for TIMP-1 as optimal cut-off values (Additional File 2: Table S2). Specifically, the number of RA-ILD^+^ patients who were positive for these defined cut-off values was 26 for MMP-2 and 29 for TIMP-1 (Fig. 4).

**Fig. 5:**
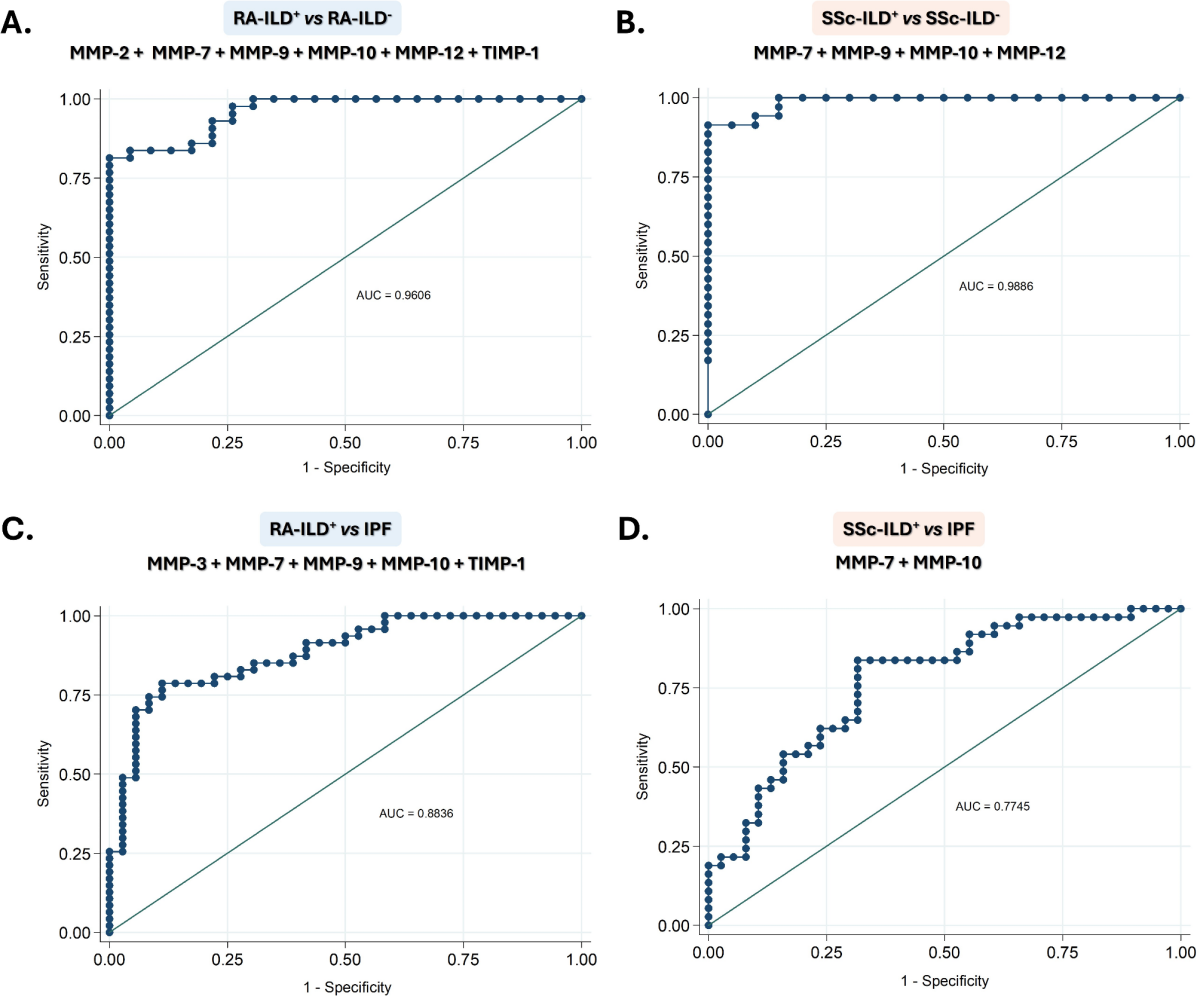
**Utility of evaluating MMPs and TIMPs together for the early and accurate diagnosis of CTDs-ILD**^**+**^ **patients.** ROC curve analysis of the combination of MMPs and TIMPs for the identification of ILD in RA patients (**A**) and SSc patients (**B**), and for the discrimination of IPF from RA-ILD^+^ (**C**) and SSc-ILD^+^ (**D**). CTDs: connective tissue diseases; ILD: interstitial lung disease; IPF: idiopathic pulmonary fibrosis; RA: rheumatoid arthritis; SSc: systemic sclerosis; MMPs: matrix metalloproteinases; TIMPs: Matrix metalloproteinases inhibitors; AUC: area under the curve

In a multivariate analysis, we found that the assessment of the combination of MMP-2, MMP-7, MMP-9, MMP-10, MMP-12, and TIMP-1 was better for the identification of ILD in RA patients than each protein independently evaluated, yielding an AUC of 0.9606 (Fig. 5A). Likewise, a combinatorial signature including MMP-7, MMP-9, MMP-10, and MMP-12 significantly increased the AUC to 0.9886 for ILD diagnosis in patients with SSc (Fig. 5B). Patients with RA-ILD^+^ and SSc-ILD^+^ who are combinatorial-marker-positive for their characterization *versus* RA-ILD^-^ and SSc-ILD^-^ patients, respectively, showed specific clinical characteristics (Additional File 3: Figure S1).

### MMPs and TIMPs constitute combinatorial biomarker signatures for the differential diagnosis of RA-ILD+ and SSc-ILD+ from IPF

**Fig. 6:**
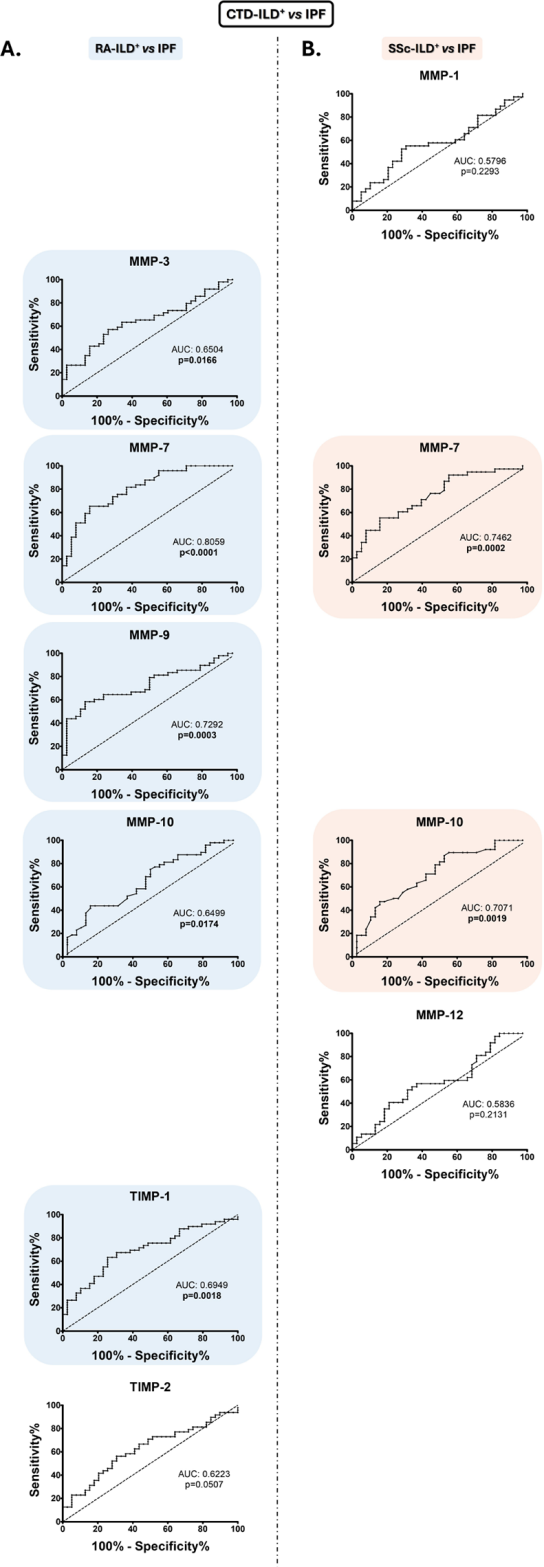
**Utility of MMPs and TIMPs as biomarkers in the differential diagnosis between CTDs-ILD**^**+**^ **and IPF patients.** ROC curve analysis of MMPs and TIMPs for the discrimination of RA-ILD^+^ from IPF (**A**) and SSc-ILD^+^ from IPF. CTDs: connective tissue diseases; ILD: interstitial lung disease; IPF: idiopathic pulmonary fibrosis; RA: rheumatoid arthritis; SSc: systemic sclerosis; MMPs: matrix metalloproteinases; TIMPs: Matrix metalloproteinases inhibitors; AUC: area under the curve. Significant results are highlighted

Notably, ROC analyses further verified the capacity of MMP-7 and MMP-10 serum levels for characterizing RA-ILD^+^*versus* IPF patients (AUC: 0.8059, *p* < 0.0001 and AUC: 0.6499, *p* = 0.0174, respectively, Fig. 6A, Additional File 2: Table S2), as well as SSc-ILD^+^*versus* IPF patients (AUC: 0.7462, *p* = 0.0002 and AUC: 0.7071, *p* = 0.0019, respectively, Fig. 6B, Additional File 2: Table S2). The optimal cut-off value for MMP-7 and MMP-10 that achieved the best sensitivity and specificity were < 35,114 pg/mL and < 567.0 pg/mL, respectively, for characterizing RA-ILD^+^*versus* IPF, and < 33,983 pg/mL and < 1007 g/mL for identifying SSc-ILD^+^*versus* IPF (Additional File 2: Table S2). In particular, the number of patients who were positive for these defined cut-off values was, in the case of RA-ILD^+^ patients, 32 for MMP-7 and 21 for MMP-10, and regarding SSc-ILD^+^ patients, 21 for MMP-7 and 32 for MMP-10 (Fig. 4).

Specifically, we confirmed the capacity of MMP-3, MMP-9, and TIMP-1 serum levels to differentiate between RA-ILD^+^ and IPF patients (AUC: 0.6504, *p* = 0.0166; AUC: 07292, *p* = 0.0003; and AUC: 0.6949, *p* = 0.0018, respectively, Fig. 6A, Additional File 2: Table S2). The optimal cut-off values were > 58,249 pg/mL for MMP-3, < 414,407 pg/mL for MMP-9, and < 57,973 pg/mL for TIMP-1 (Additional File 2: Table S2). Specifically, the number of RA-ILD^+^ patients who were positive for these defined cut-off values was 28 for MMP-3, 28 for MMP-9, and 31 for TIMP-1 (Fig. 4).

Although we found differences in TIMP-2 levels between RA-ILD^+^ and IPF patients, this protein did not present the necessary capacity to distinguish between these diseases (*p* = 0.0507, Fig. 6A, Additional File 2: Table S2). Equally, we observed by ROC analyses that MMP-1 and MMP-12 were not helpful as diagnostic tools in the differential diagnosis of SSc-ILD^+^ and IPF (*p* = 0.2293 and *p* = 0.2131, respectively, Fig. 6B, Additional File 2: Table S2).

In a further step, we disclosed that a 5-biomarker signature consisting of MMP-3, MMP-7, MMP-9, MMP-10, and TIMP-1 effectively better-characterized RA-ILD^+^*versus* IPF compared to when independently assessed, yielding an AUC of 0.8836 (Fig. 5C). Likewise, the multivariable analyses indicated that the combined evaluation of MMP-7 and MMP-10 was better for characterizing SS-ILD^+^*versus* IPF (AUC: 0.7745, Fig. 5D). Patients with RA-ILD^+^ and SSc-ILD^+^ who are combinatorial-marker-positive for their characterization *versus* IPF patients showed specific clinical characteristics (Additional File 3: Figure S1).

### MMP-10 serum levels are related to the RA-ILD+ severity

Higher MMP-10 serum levels were associated with worse baseline pulmonary function in RA-ILD^+^ patients represented by decreased forced vital capacity (FVC) and forced expiratory volume in one second (FEV1) (*r*=-0.3483, *p* = 0.0205 and *r*=-0.3556, *p* = 0.0178, respectively, Additional File 4: Figure S2). However, no significant correlation was found between the rest of the MMPs and TIMPs studied and PFTs in RA-ILD^+^ and SSc-ILD^+^ patients (data not shown). Besides, levels of MMPs and TIMPs in RA-ILD^+^ and SSc-ILD^+^ patients were unrelated to the radiological pattern more serious such as the usual interstitial pneumonia (UIP) pattern (data not shown). Similarly, RA-ILD^+^ and SSc-ILD^+^ patients with progressive pulmonary fibrosis were not different concerning MMPs and TIMPs from their counterparts (data not shown).

Finally, we found that RA-ILD^+^ and SSc-ILD^+^ patients who had higher MMP and TIMP levels than the optimal cut-off previously defined for their discrimination from IPF (the worst prognostic ILD), did not predict poor survival (time to death or lung transplant) (Additional File 5: Figure S3, and Additional File 6: Fig. S4, respectively).

### MMP-2 and TIMP-1 serum levels are associated with RA-ILD+ clinical features

TIMP-1 serum levels were lower in rheumatoid factor positive RA-ILD^+^ patients than in their counterparts (49426.793 pg/mL ± 17870.765 pg/mL vs. 73250.944 pg/mL ± 24312.161 pg/mL, *p* = 0.002). Furthermore, patients with RA-ILD^+^ undergoing conventional synthetic disease-modifying anti-rheumatic drugs (csDMARDs) therapy showed decreased levels of MMP-2 compared to those not treated with csDMARDs (155844.92 pg/mL ± 27590.166 pg/mL vs. 186015.17 pg/mL ± 30834.722 pg/mL, *p* < 0.001). However, no relationship with anti-cyclic citrullinated peptide antibodies (ACPA) status, ILD and CTD duration, and biologic disease-modifying anti-rheumatic drugs (bDMARDs) was found in RA-ILD^+^ patients for any of the proteins evaluated (MMP-2, MMP-7, MMP-9, MMP-10, MMP-12, and TIMP-1) (data not shown).

Similarly, in patients with SSc-ILD^+^, MMP-3, MMP-7, MMP-9, MMP-10, and TIMP-1 levels were not associated with antibody positivity status (anti-nuclear antibodies, anti-centromere antibodies and anti-topoisomerase I antibodies), ILD and CTD duration, received treatments (csDMARDs, bDMARDs, and vasodilators), or the presence of other SSc clinical characteristic (renal impairment, cardiac involvement, Raynaud’s phenomenon, esophageal dysfunction, calcinosis, and synovitis) (data not shown).

## Discussion

There are significant gaps in the understanding of CTDs-ILD that hinder their early identification and lead to underdiagnosis of the disease. The limitations of currently available clinical tools highlight the urgent need to complement them with accessible and non-invasive diagnostic methods. Finding biomarkers would improve CTD-ILD detection, thereby preventing irreversible damage and reducing the associated mortality in these patients. In this context, the abnormal remodeling of ECM is a hallmark of lung fibrosis, but the role of MMPs and TIMPs in the pathogenesis of CTDs-ILD has not been fully elucidated. Accordingly, we focused on identifying useful MMPs and TIMPs as new biomarkers with clinical value in the diagnosis and the prognosis of ILD in the most affected CTD for this devastating disease, SSc and RA.

**Fig. 7:**
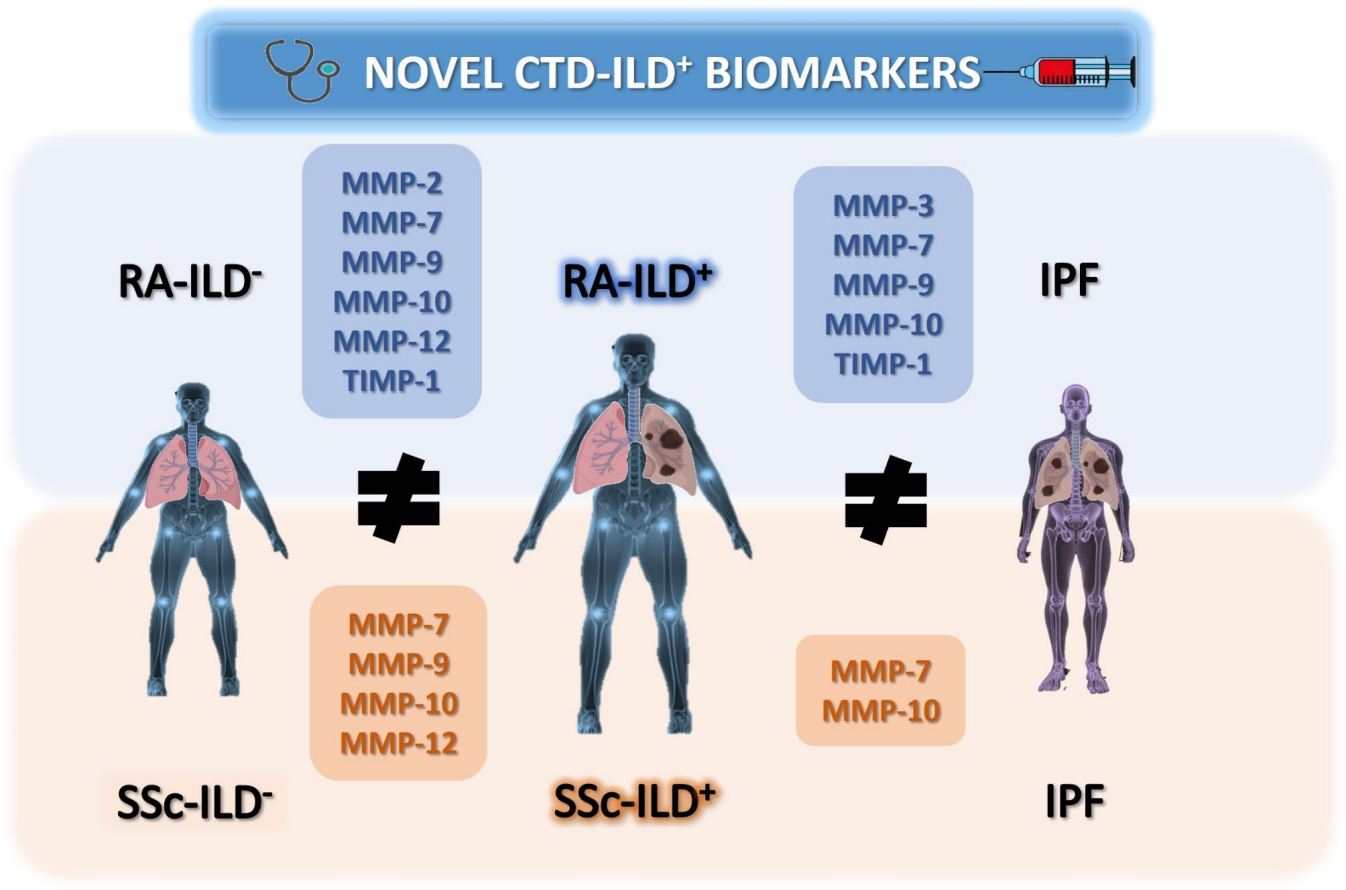
**MMPs and TIMPs as promising biomarkers in the early and accurate diagnosis of CTDs-ILD**^**+**^. MMPs and TIMPs found as potential candidates for the discrimination of CTDs-ILD^+^ from CTDs-ILD^−^ as well as CTDs-ILD^+^ and IPF. CTDs: connective tissue diseases; ILD: interstitial lung disease; IPF: idiopathic pulmonary fibrosis; RA: rheumatoid arthritis; SSc: systemic sclerosis; MMPs: matrix metalloproteinases; TIMPs: Matrix metalloproteinases inhibitors

To the best of our knowledge, we demonstrate for the first time that models consisting of the combination of remodeling ECM-biomarkers are strongly associated with the presence of CTD-ILD^+^, both RA-ILD^+^ and SSc-ILD^+^ (Fig. 7).

On the one hand, the early detection of ILD in both SSc and RA is vital to improve the quality of life of patients, to avoid a poor prognosis, and to reduce their mortality rates. To facilitate the CTD-ILD early diagnosis, we discovered that the combination of MMP-2, MMP-7, MMP-9, MMP-10, MMP-12, and TIMP-1 can characterize reliably RA-ILD^+^ patients *versus* those RA patients without ILD. Consistent with our results, previous studies point to MMP-7 as a key biomarker of RA-ILD^+^ showing higher concentrations of this protein in these patients in relation to those with RA-ILD^−^ (Luedders et al. 2024; Kass et al. 2020; Chen et al. 2015; Doyle et al. 2015). Recently, shifts in MMP-2, MMP-9, and MMP-10 levels have also been associated with the presence of ILD in RA patients by Kass et al. (2020) (Kass et al. 2020). Therefore, our findings confirmed the crucial role of MMP-2, MMP-7, MMP-9, and MMP-10 in RA-ILD^+^ and clarified that these together with two new relevant proteins, MMP-12 and TIMP-1, provide a useful combinatorial biomarker signature for the early diagnosis of ILD in patients with RA. Interestingly, some of these MMPs also remained relevant in our analyses of SSc, which demonstrated that a combinatorial signature including MMP-7, MMP-9, MMP-10, and MMP-12 was robustly associated with the presence of ILD in SSc patients. On this line, a sound body of evidence considers that an excess of MMP-7 may serve as a marker for pulmonary impairment and progressive disease in SSc patients (Györfi et al. 2024; Chen and Chen 2020; Bonhomme et al. 2019; Moinzadeh et al. 2011), further supporting our results. Although fewer studies have been conducted on other MMPs, some reports also reached the same results as ours showing elevated levels of MMP-9 (Guiot et al. 2020; Kim et al. 2005) and MMP-12 (Bonhomme et al. 2019; Manetti et al. 2012) in patients with SSc-ILD^+^. Notably, the findings emerging from our work revealed MMP-10 as a novel biomarker of SSc-ILD^+^, and its combination with MMP-7, MMP-9, and MMP-12 establishes a biomarker signature that significantly improves the ability to identify ILD in SSc patients. In general, it is plausible to think that the increment of MMP-2, MMP-7, MMP-9, MMP-10, and MMP-12 and the depletion of TIMP-1 observed in our patients with CTD-ILD^+^ leads to an increase in ECM degradation that affects the adherence of cells to the ECM and triggers the release of cytokines, inflammatory mediators, and growth factors that were trapped in it (such as vascular endothelial growth factor and transforming growth factor-β), providing profibrotic signals from the microenvironment to cells. This is in line with the current notion postulating that MMPs and TIMPs are involved in the pathological remodeling ECM characteristic of lung fibrosis, not only by their imbalance but also by acting as key molecules in the cross-talk between the ECM and cells, which could influence the biological process of ILD (Vandenbroucke et al. 2011; Lv et al. 2022; Zhou et al. 2024; Gueders et al. 2006).

On the other hand, distinguishing CTD-ILD^+^ from IPF patients has important prognostic and therapeutic implications for both RA-ILD^+^ and SSc-ILD^+^ patients. To address this concern, our study provided a novel 5-biomarker signature consisting of MMP-3, MMP-7, MMP-9, MMP-10, and TIMP-1 that effectively characterized RA-ILD^+^*versus* IPF. Remarkably, we observed that the combined MMP-7 and MMP-10 assessment allowed for better characterization of SSc-ILD^+^*versus* IPF. There is little information on this topic (Kass et al. 2020; White et al. 2016) since most MMPs and TIMPs studies performed in ILD focused exclusively on their role in the development of IPF. In this setting, MMP-7 (Atkinson and Senior 2003; Balci et al. 2023; Inoue et al. 2020; Craig et al. 2015; Pardo and Selman 2006; Richards et al. 2012; Rosas et al. 2008; Song et al. 2013; Todd et al. 2020; Tzouvelekis et al. 2017; Wang et al. 2023a) and MMP-9 (Craig et al. 2015; Pardo and Selman 2006; Todd et al. 2020; Wang et al. 2023a) are the best-reported MMPs and have been postulated as the determining proteins in the development of active fibrosis and the prediction of poor overall survival in IPF. Nevertheless, other reports have described the role of MMP-3 (Wang et al. 2023a) and TIMP-1 (Todd et al. 2020) in IPF pathogenesis. Importantly, our discoveries further demonstrated the role of these MMPs and TIMPs in lung fibrosis, including MMP-10 as a new contributor, and revealed, for the first time, variation in their levels depending on the type of ILD. We speculate that the higher levels of MMP-7, MMP-9, and MMP-10 found in our IPF patients compared to those with CTD-ILD^+^ may describe a greater ECM degradation in IPF, which makes sense considering that it is the most severe and the worst prognostic ILD based largely on the lung fibrotic process. Regarding MMP-3, this is one of the most critical proteases in the process of cartilage degradation in RA (Skacelova et al. 2017; Lerner et al. 2018). Hence, the higher levels of MMP-3 found in our RA-ILD^+^ patients compared to those with IPF could be explained by the influence of both lung fibrosis and the inflammatory activity of RA. Moreover, and in agreement with our results, accumulating evidence indicates the importance of TIMP activities in the fibrosis progression of several pathological conditions (Kim et al. 2005), reporting increased TIMP-1 levels in IPF patients (Todd et al. 2020). The highest TIMP-1 levels discovered in our IPF patients could also be supported by its ability to exert diverse biological functions independent of their ability to inhibit metalloproteinases (Costanzo et al. 2022).

Lastly, detecting CTD-ILD^+^ patients progressing to exacerbated disease is crucial to prevent irreversible lung damage through the early application of appropriate therapy. Interestingly, we noticed that MMP-10 was related to the severity of RA-ILD^+^ patients characterized by a worse baseline pulmonary function. Previous reports have already associated exacerbation and prognosis of RA-ILD^+^ and SSc-ILD^+^ with MMPs, emphasizing MMP-7 (Chen et al. 2023; Chen and Chen 2020; Moinzadeh et al. 2011), MMP-9 (Lv et al. 2022; Luedders et al. 2024; Guiot et al. 2020) and MMP-12 (Manetti et al. 2012). Although we did not find further MMPs or TIMPs associated with the severity or poor evolution of both RA-ILD^+^ and SSc-ILD^+^, in agreement with other authors (Chen et al. 2022), our work showed for the first time that serum MMP-10 levels may have clinical value in screening the progression of severe phases of RA-ILD^+^ patients. In addition, and for the rest of the proteins studied, relationships with other clinical features of RA-ILD^+^ patients were found, observing associations of treatment and antibody status with levels of MMP-2 and TIMP-1, respectively.

In summary, our study revealed novel ECM remodeling-biomarkers signatures in peripheral blood that represent a viable approach to developing clinically useful tools capable of early and accurate identification of RA-ILD^+^ and SSc-ILD^+^ patients. The implementation of these biomarkers together with current diagnostic tools in clinical practice would constitute a great advance for earlier and more effective management of this challenging entity, ultimately leading to decreased lung damage and better outcomes for these patients.

## Conclusion

MMPs and TIMPs form combinatorial biomarker signatures with clinical value for non-invasive, early, and accurate diagnosis of RA-ILD^+^ and SSc-ILD^+^, constituting promising screening tools in clinical practice.

## Electronic supplementary material

Below is the link to the electronic supplementary material.

**Additional File 1: Table S1. **Demographic and clinical characteristics of all the patients of the study.

**Additional File 2: Table S2**. ROC curves analysis for the discrimination of CTDs-ILD^+^ from CTDs-ILD^-^ and CTDs-ILD^+^ from IPF.

**Additional File 3: Figure S1. Clinical characteristics of patients with RA-ILD**^**+**^ **and SSc-ILD**^**+**^ **who are combinatorial-marker-positive for their characterization *versus*** **patients with RA-ILD**^**-**^ **and SSc-ILD**^**-**^**, respectively, and idiopathic pulmonary fibrosis patients. **RA: rheumatoid arthritis; ILD: interstitial lung disease; HRCT: high resolution computed tomography; UIP: usual interstitial pneumonia; NSIP: non-specific interstitial pneumonia; FVC: forced vital capacity; FEV1: forced expiratory volume in one second; DLCO: diffusing capacity of the lung for carbon monoxide; RF: rheumatoid factor; ACPA: anti-cyclic citrullinated peptide antibodies; SSc: systemic sclerosis; ANA: anti-nuclear antibodies; ACA: anti-centromere antibodies; ATA: anti-topoisomerase I antibodies.

**Additional File 4: Figure S2. Relationship of MMP-10 serum levels with FVC (A) and FEV1 (B) in patients with RA-ILD**^**+**^**. **MMP: matrix metalloproteinase; FVC: forced vital capacity; FEV1: forced expiratory volume in one second; RA: rheumatoid arthritis; ILD: interstitial lung disease. Significant results are highlighted.

Additional File 5: Figure S3. Survival (time to death or lung transplantation) according to the serum level* of MMP-3 (A), MMP-1 (B), MMP-9 (C), MMP-10 (D), and TIMP-1 (E) in patients with RA-ILD^**+**^**. **RA: rheumatoid arthritis; ILD: interstitial lung disease; MMPs: matrix metalloproteinases; TIMPs: Matrix metalloproteinases inhibitors. *The cut-off of MMPs and TIMP levels was determined by ROC curves for discriminating RA-ILD^+^ and idiopathic pulmonary fibrosis.

Additional File 6: Figure S4. Survival (time to death or lung transplantation) according to the serum level* of MMP-7 (A) and MMP-10 (B) in patients with SSc-ILD^**+**^**. **SSc: systemic sclerosis; ILD: interstitial lung disease; MMPs: matrix metalloproteinases; TIMPs: Matrix metalloproteinases inhibitors. *The cut-off of MMP levels was determined by receiver operating characteristic curves for discriminating SSc-ILD^+^ and idiopathic pulmonary fibrosis.

## Data Availability

No datasets were generated or analysed during the current study.

## Abbreviations

ACA: anti-centromere antibodies
ACPA: anti-cyclic citrullinated peptide antibodies
ACR: American College of Rheumatology
ANA: anti-nuclear antibodies
ATA: anti-topoisomerase I antibodies
ATS: American Thoracic Society
ANOVA: analysis of variance
AUC: area under the curve
bDMARDs: biologic disease-modifying anti-rheumatic drugs
CI: confident interval
csDMARDs: conventional synthetic disease-modifying anti-rheumatic drugs
CTDs: connective tissue diseases
ECM: extracellular matrix
ERS: European Respiratory Society
EULAR: European League Against Rheumatism
FEV1: forced expiratory volume in one second
FVC: forced vital capacity
HRCT: high-resolution computed tomography
ILD: interstitial lung disease
IPF: idiopathic pulmonary fibrosis
MMPs: matrix metalloproteinases
PFTs: pulmonary function tests
RA: rheumatoid arthritis
ROC: receiver operating characteristic
SD: standard deviation
SSc: systemic sclerosis
TIMPs: tissue inhibitors of metalloproteinases
UIP: usual interstitial pneumonia

## References

[CR7] Ahmed S, Handa R. Management of connective tissue disease–related interstitial lung disease. Curr Pulmonol Rep. 2022;11:86–98. 35530438 10.1007/s13665-022-00290-w PMC9062859

[CR31] Aletaha D, Neogi T, Silman AJ, Funovits J, Felson DT, Bingham CO, et al. 2010 rheumatoid arthritis classification criteria: an American College of Rheumatology/European League against Rheumatism collaborative initiative. Arthritis Rheum. 2010;62:2569–81. 20872595 10.1002/art.27584

[CR15] Atkinson JJ, Senior RM. Matrix metalloproteinase-9 in lung remodeling. Am J Respir Cell Mol Biol. 2003;28:12–24. 12495928 10.1165/rcmb.2002-0166TR

[CR19] Balci A, Düz ME, Vurmaz A, Çilekar Ş, Kaya F. Comprehensive biomarker analysis of patients with idiopathic pulmonary fibrosis and interstitial lung disease with healthy individuals. Eur Rev Med Pharmacol Sci. 2023;27:5468–79. 37401283 10.26355/eurrev_202306_32783

[CR42] Bonhomme O, André B, Gester F, De Seny D, Moermans C, Struman I, et al. Biomarkers in systemic sclerosis-associated interstitial lung disease: review of the literature. Rheumatology (Oxford). 2019;58:1534–46. 31292645 10.1093/rheumatology/kez230 PMC6736409

[CR18] Brew K, Dinakarpandian D, Nagase H. Tissue inhibitors of metalloproteinases: evolution, structure and function. Biochim Biophys Acta. 2000;1477:267–83. 10708863 10.1016/s0167-4838(99)00279-4

[CR2] Cerro Chiang G, Parimon T. Understanding interstitial Lung diseases Associated with connective tissue disease (CTD-ILD): Genetics, Cellular Pathophysiology, and Biologic drivers. Int J Mol Sci. 2023;24:2405. 36768729 10.3390/ijms24032405 PMC9917355

[CR41] Chen ZX, Chen W. Matrix metalloproteinase 7 is a candidate biomarker in systemic sclerosis-associated interstitial lung disease. Acta Reumatol Port. 2020;45:191–200. 33139675

[CR38] Chen J, Doyle TJ, Liu Y, Aggarwal R, Wang X, Shi Y, et al. Biomarkers of rheumatoid arthritis-associated interstitial lung disease. Arthritis Rheumatol. 2015;67:28–38. 25302945 10.1002/art.38904 PMC4624107

[CR54] Chen J, Chen Y, Liu D, Lin Y, Zhu L, Song S, et al. Predictors of long-term prognosis in rheumatoid arthritis-related interstitial lung disease. Sci Rep. 2022;12:9469. 35676424 10.1038/s41598-022-13474-w PMC9177673

[CR29] Chen H, Tang J, Liang J, Huang D, Pan C, Liu S, et al. Clinical association study on the matrix metalloproteinase expression in the serum of patients with connective tissue disease complicated with interstitial lung disease. Arch Rheumatol. 2023;38:367–74. 38046255 10.46497/ArchRheumatol.2023.9547 PMC10689012

[CR14] Chuliá-Peris L, Carreres-Rey C, Gabasa M, Alcaraz J, Carretero J, Pereda J. Matrix metalloproteinases and their inhibitors in pulmonary fibrosis: EMMPRIN/CD147 comes into play. Int J Mol Sci. 2022;23:6894. 35805895 10.3390/ijms23136894 PMC9267107

[CR53] Costanzo L, Soto B, Meier R, Geraghty P. The Biology and Function of Tissue Inhibitor of Metalloproteinase 2 in the lungs. Pulm Med. 2022;2022:2022. 10.1155/2022/3632764 PMC9825218 36624735

[CR21] Craig VJ, Zhang L, Hagood JS, Owen CA. Matrix metalloproteinases as therapeutic targets for idiopathic pulmonary fibrosis. Am J Respir Cell Mol Biol. 2015;53:585–600. 26121236 10.1165/rcmb.2015-0020TR PMC4742954

[CR39] Doyle TJ, Patel AS, Hatabu H, Nishino M, Wu G, Osorio JC, et al. Detection of rheumatoid arthritis-interstitial lung disease is enhanced by serum biomarkers. Am J Respir Crit Care Med. 2015;191:1403–12. 25822095 10.1164/rccm.201411-1950OC PMC4476561

[CR4] Enomoto N. Relationship between idiopathic interstitial pneumonias (IIPs) and connective tissue disease-related interstitial lung disease (CTD-ILD): a narrative review. Respir Investig. 2024;62:465–80. 38564878 10.1016/j.resinv.2024.03.006

[CR48] Gueders MM, Foidart JM, Noel A, Cataldo DD. Matrix metalloproteinases (MMPs) and tissue inhibitors of MMPs in the respiratory tract: potential implications in asthma and other lung diseases. Eur J Pharmacol. 2006;533:133–44. 16487964 10.1016/j.ejphar.2005.12.082

[CR44] Guiot J, Henket M, Andre B, Herzog M, Hardat N, Njock MS, et al. A new nucleosomic-based model to identify and diagnose SSc-ILD. Clin Epigenetics. 2020;12:124. 32807242 10.1186/s13148-020-00915-4 PMC7430109

[CR1] Guiot J, Miedema J, Cordeiro A, De Vries-Bouwstra JK, Dimitroulas T, Søndergaard K, et al. Practical guidance for the early recognition and follow-up of patients with connective tissue disease-related interstitial lung disease. Autoimmun Rev. 2024;23:103582. 39074630 10.1016/j.autrev.2024.103582

[CR40] Györfi AH, Filla T, Dickel N, Möller F, Li YN, Bergmann C, et al. Performance of serum biomarkers reflective of different pathogenic processes in systemic sclerosis-associated interstitial lung disease. Rheumatology (Oxford). 2024;63:962–9. 37421394 10.1093/rheumatology/kead332

[CR9] Hoffmann-Vold AM, Fretheim H, Meier C, Maurer B. Circulating biomarkers of systemic sclerosis– interstitial lung disease. J Scleroderma Relat Disord. 2020;5 2suppl:41–7. 35382223 10.1177/2397198319894851 PMC8922568

[CR20] Inoue Y, Kaner RJ, Guiot J, Maher TM, Tomassetti S, Moiseev S, et al. Diagnostic and prognostic biomarkers for chronic Fibrosing interstitial lung diseases with a progressive phenotype. Chest. 2020;158:646–59. 32268131 10.1016/j.chest.2020.03.037

[CR37] Kass DJ, Nouraie M, Glassberg MK, Ramreddy N, Fernandez K, Harlow L, et al. Comparative profiling of serum protein biomarkers in Rheumatoid Arthritis–Associated interstitial lung Disease and Idiopathic Pulmonary Fibrosis. Arthritis Rheumatol. 2020;72:409–19. 31532072 10.1002/art.41123

[CR45] Kim W-U, Min S-Y, Cho M-L, Hong K-H, Shin Y-J, Park S-H, et al. Elevated matrix metalloproteinase-9 in patients with systemic sclerosis. Arthritis Res Ther. 2005;7:R71–9. 15642145 10.1186/ar1454 PMC1064883

[CR17] Klein T, Bischoff R. Physiology and pathophysiology of matrix metalloproteases. Amino Acids. 2011;41:271–90. 20640864 10.1007/s00726-010-0689-x PMC3102199

[CR52] Lerner A, Neidhöfer S, Reuter S, Matthias T. MMP3 is a reliable marker for disease activity, radiological monitoring, disease outcome predictability, and therapeutic response in rheumatoid arthritis. Best Prac Res Clin Rheumatol. 2018;32:550–62. 10.1016/j.berh.2019.01.006 31174824

[CR36] Luedders BA, Wheeler AM, Ascherman DP, Baker JF, Duryee MJ, Yang Y, et al. Plasma matrix metalloproteinase concentrations and risk of interstitial lung disease in a prospective rheumatoid arthritis cohort. Arthritis Rheumatol. 2024;76:1013–22. 38268499 10.1002/art.42812 PMC11213673

[CR30] Lv C, Zhang Q, Tang P, Guo L, Ding Q, Serum. MMP-9, SP-D, and VEGF levels reflect the severity of connective tissue disease-associated interstitial lung diseases. Adv Rheumatol. 2022;62:37. 36303230 10.1186/s42358-022-00269-w

[CR34] Lynch DA, Sverzellati N, Travis WD, Brown KK, Colby TV, Galvin JR, et al. Diagnostic criteria for idiopathic pulmonary fibrosis: a Fleischner Society White Paper. Lancet Respir Med. 2018;6:138–53. 29154106 10.1016/S2213-2600(17)30433-2

[CR46] Manetti M, Guiducci S, Romano E, Bellando-Randone S, Conforti ML, Ibba-Manneschi L, et al. Increased serum levels and tissue expression of matrix metalloproteinase-12 in patients with systemic sclerosis: correlation with severity of skin and pulmonary fibrosis and vascular damage. Ann Rheum Dis. 2012;71:1064–72. 22258486 10.1136/annrheumdis-2011-200837

[CR3] Matson SM, Demoruelle MK. Connective tissue Disease Associated interstitial lung disease. Rheum Dis Clin North Am. 2024;50:423–38. 38942578 10.1016/j.rdc.2024.03.001

[CR43] Moinzadeh P, Krieg T, Hellmich M, Brinckmann J, Neumann E, Müller-Ladner U, et al. Elevated MMP-7 levels in patients with systemic sclerosis: correlation with pulmonary involvement. Exp Dermatol. 2011;20:770–3. 21707759 10.1111/j.1600-0625.2011.01321.x

[CR32] n Den Hoogen F, Khanna D, Fransen J, Johnson SR, Baron M, Tyndall A, et al. Arthritis & Rheumatism 2013 classification criteria for systemic sclerosis. Arthritis Rheum. 2013;65:2737–47. 24122180 10.1002/art.38098 PMC3930146

[CR22] Pardo A, Selman M. Matrix metalloproteases in aberrant fibrotic tissue remodeling. Proc Am Thorac Soc. 2006;3:383–8. 16738205 10.1513/pats.200601-012TK

[CR28] Peng Z, Konai MM, Avila-Cobian LF, Wang M, Mobashery S, Chang M. MMP-1 and ADAM10 as targets for therapeutic intervention in idiopathic pulmonary fibrosis. ACS Pharmacol Transl Sci. 2022;5:548–54. 35983283 10.1021/acsptsci.2c00050 PMC9380212

[CR11] Pulito-Cueto V, Remuzgo-Martínez S, Genre F, Atienza-Mateo B, Mora-Cuesta VM, Iturbe-Fernández D, et al. Elevated VCAM-1, MCP-1 and ADMA serum levels related to pulmonary fibrosis of interstitial lung disease associated with rheumatoid arthritis. Front Mol Biosci. 2022;9:1056121. 36601584 10.3389/fmolb.2022.1056121 PMC9806218

[CR10] Pulito-Cueto V, Genre F, López-Mejías R, Mora-Cuesta VM, Iturbe-Fernández D, Portilla V, et al. Endothelin-1 as a biomarker of idiopathic pulmonary fibrosis and interstitial lung Disease Associated with Autoimmune diseases. Int J Mol Sci. 2023a;24:1275. 36674789 10.3390/ijms24021275 PMC9862125

[CR12] Pulito-Cueto V, Remuzgo-Martínez S, Genre F, Atienza-Mateo B, Mora-Cuesta VM, Iturbe-Fernández D, et al. E-Selectin, ICAM-1, and ET-1 biomarkers address the concern of the Challenging diagnosis of interstitial lung disease in patients with Autoimmune diseases. Int J Mol Sci. 2023b;24:12518. 37569893 10.3390/ijms241512518 PMC10420063

[CR35] Raghu G, Remy-Jardin M, Richeldi L, Thomson CC, Antoniou KM, Bissell BD, et al. Idiopathic pulmonary fibrosis (an update) and progressive pulmonary fibrosis in adults: an Official ATS/ERS/JRS/ALAT Clinical Practice Guideline. Am J Respir Crit Care Med. 2022;205:E18–47. 35486072 10.1164/rccm.202202-0399ST PMC9851481

[CR23] Richards TJ, Kaminski N, Baribaud F, Flavin S, Brodmerkel C, Horowitz D, et al. Peripheral blood proteins predict mortality in idiopathic pulmonary fibrosis. Am J Respir Crit Care Med. 2012;185:67–76. 22016448 10.1164/rccm.201101-0058OC PMC3262037

[CR24] Rosas IO, Richards TJ, Konishi K, Zhang Y, Gibson K, Lokshin AE, et al. MMP1 and MMP7 as potential peripheral blood biomarkers in idiopathic pulmonary fibrosis. PLoS Med. 2008;5:e93. 18447576 10.1371/journal.pmed.0050093 PMC2346504

[CR51] Skacelova M, Hermanova Z, Horak P, Kazi A, Langova K. Higher levels of matrix metalloproteinase-3 in patients with RA reflect disease activity and structural damage. Biomed Pap Med Fac Univ Palacky Olomouc Czech Repub. 2017;161:296–302. 28461705 10.5507/bp.2017.015

[CR25] Song JW, Do KH, Jang SJ, Colby TV, Han S, Kim DS. Blood biomarkers MMP-7 and SP-A: predictors of outcome in idiopathic pulmonary fibrosis. Chest. 2013;143:1422–9. 23715088 10.1378/chest.11-2735

[CR8] Stainer A, Tonutti A, De Santis M, Amati F, Ceribelli A, Bongiovanni G, et al. Unmet needs and perspectives in rheumatoid arthritis-associated interstitial lung disease: a critical review. Front Med (Lausanne). 2023;10:1129939. 37007765 10.3389/fmed.2023.1129939 PMC10062456

[CR26] Todd JL, Vinisko R, Liu Y, Neely ML, Overton R, Flaherty KR, et al. Circulating matrix metalloproteinases and tissue metalloproteinase inhibitors in patients with idiopathic pulmonary fibrosis in the multicenter IPF-PRO Registry cohort. BMC Pulm Med. 2020;20:64. 32171287 10.1186/s12890-020-1103-4 PMC7071646

[CR33] Travis WD, Costabel U, Hansell DM, King TE, Lynch DA, Nicholson AG, et al. An official American Thoracic Society/European Respiratory Society Statement: update of the International Multidisciplinary classification of the idiopathic interstitial pneumonias. Am J Respir Crit Care Med. 2013;188:733–48. 24032382 10.1164/rccm.201308-1483ST PMC5803655

[CR27] Tzouvelekis A, Herazo-Maya JD, Slade M, Chu JH, Deiuliis G, Ryu C, et al. Validation of the prognostic value of MMP-7 in idiopathic pulmonary fibrosis. Respirology. 2017;22:486–93. 27761978 10.1111/resp.12920 PMC5352520

[CR16] Vandenbroucke RE, Dejonckheere E, Libert C. A therapeutic role for matrix metalloproteinase inhibitors in lung diseases? Eur Respir J. 2011;38:1200–14. 21659416 10.1183/09031936.00027411

[CR5] Wang Q, Xie Z, Wan N, Yang L, Jin Z, Jin F, et al. Potential biomarkers for diagnosis and disease evaluation of idiopathic pulmonary fibrosis. Chin Med J (Engl). 2023a;136:1278–90. 37130223 10.1097/CM9.0000000000002171 PMC10309524

[CR13] Wang Y, Jiao L, Qiang C, Chen C, Shen Z, Ding F, et al. The role of matrix metalloproteinase 9 in fibrosis diseases and its molecular mechanisms. Biomed Pharmacother. 2024;171:116116. 38181715 10.1016/j.biopha.2023.116116

[CR49] White ES, Xia M, Murray S, Dyal R, Flaherty CM, Flaherty KR, et al. Plasma surfactant protein-D, matrix metalloproteinase-7, and osteopontin index distinguishes idiopathic pulmonary fibrosis from other idiopathic interstitial pneumonias. Am J Respir Crit Care Med. 2016;194:1242–51. 27149370 10.1164/rccm.201505-0862OC PMC5114439

[CR47] Zhou X, Zhang C, Yang S, Yang L, Luo W, Zhang W, et al. Macrophage-derived MMP12 promotes fibrosis through sustained damage to endothelial cells. J Hazard Mater. 2024;461:132733. 37816293 10.1016/j.jhazmat.2023.132733

[CR6] Zhu W, Liu C, Tan C, Zhang J. Predictive biomarkers of disease progression in idiopathic pulmonary fibrosis. Heliyon. 2023;10:e23543. 38173501 10.1016/j.heliyon.2023.e23543 PMC10761784

