# Supplementary material for: Matrix metalloproteinases and their tissue inhibitors as upcoming biomarker signatures of connective tissue diseases-related interstitial lung disease: towards an earlier and accurate diagnosis
Source: Mol Med. 2025 Feb 20;31:70. doi: 10.1186/s10020-025-01128-2 (PMC11844142; doi:10.1186/s10020-025-01128-2)
Supplement: Supplementary file 1 — Additional File 1: Table S1. Demographic and clinical characteristics of all the patients of the study. [file 10020_2025_1128_MOESM1_ESM.pdf]

Table S1. Demographic and clinical characteristics of all the patients of the study.

	Study Objective Groups		*Comparative Groups*		
	RA-ILD⁺	***SSc-ILD⁺***	***RA-ILD⁻***	***SSc-ILD⁻***	***IPF***
	n=49	***n=38***	***n=25***	***n=20***	***n=39***
Sex (women), n (%)	21 (42.9)	23 (60.5)	15 (60.0)	18 (90.0)	7 (17.9)
Age at study (years), mean ± SD	66,6 ± 9.3	57,3 ± 9.3	60.1 ± 11.8	56.6 ± 15.4	65.7 ± 8.5
Smoking ever, n (%)	37 (75.5)	20 (52.6)	13 (52.0)	11 (55.0)	33 (84.6)
CRP (mg/dL), mean ± SD	1.1 ± 1.4	0.68 ± 1.2	0.5 ± 0.5	0.5 ± 0.5	-
ESR (mm/1st hour), mean ± SD	27.1 ± 23.6	22.7 ± 15.9	14.4 ± 12.4	17.2 ± 13.4	-
**Antibody status**					
RF+, n (%)	41 (83.7)	-	11 (44.0)	-	-
ACPA+, n (%)	43 (87.8)	-	15 (60.0)	-	-
ANA+, n (%)	-	37 (97.4)	-	18 (90.0)	-
ACA+, n (%)	-	3 (7.9)	-	9 (45.0)	-
ATA (anti-Scl70) +, n (%)	-	17 (44.7)	-	4 (20.0)	-
**CTD duration (years)**, mean ± SD	7.6 ± 8.9	6.5 ± 8.3	3.65 ± 7.5	9.6 ± 8.1	-
**ILD duration (years)**, mean ± SD	2.6 ± 3.1	5.3 ± 7.5	-	-	3.4 ± 3.4
**Pulmonary function tests**					
FVC (% predicted), mean ± SD	85.8 ± 24.9	77.2 ± 23.4	99.2 ± 16.0	106.6 ± 15.9	81.9 ± 18.1
FEV1 (% predicted), mean ± SD	84.7 ± 22.1	76.2 ± 22.4	94.9 ± 22.0	101.9 ± 17.8	81.6 ± 19.2
DLCO (% predicted), mean ± SD	44.1 ± 18.3	45.1 ± 19.9	79.9 ± 20.0	71.5 ± 15.3	34.2 ± 17.3
**HRCT**					
Pulmonary involvement in HRCT, n (%)	49 (100.0)	38 (100.0)	0 (0.0)	0 (0.0)	39 (100.0)
UIP pattern, n (%)	26 (53.1)	8 (21.0)	-	-	39 (100.0)
Probable UIP pattern, n (%)	7 (14.3)	2 (5.3)	-	-	0 (0.0)
Indeterminate for UIP pattern, n (%)	1 (2.0)	1 (2.6)			
NSIP pattern, n (%)	13 (26.5)	24 (63.2)	-	-	0 (0.0)
Non-NSIP pattern, n (%)	2 (4.1)	3 (7.9)	-	-	0 (0.0)
**Progressive pulmonary fibrosis**, n (%)	10 (20.4)	10 (26.3)	-	-	39 (100)
**Treatments**					
csDMARDs, n (%)	23 (46.9)	27 (71.1)	13 (52.0)	12 (60.0)	-
bDMARDs, n (%)	20 (40.8)	11 (28.9)	3 (12.0)	2 (10.0)	-
Vasodilators, n (%)	-	19 (50.0)	-	14 (70.0)	-
**Other SSc clinical manifestations**					

Renal impairment, n (%)	-	2 (5.3)	-	1 (5.0)	-
Cardiac involvement, n (%)	-	6 (15.8)	-	1 (5.0)	-
Raynaud's phenomenon, n (%)	-	33 (86.8)	-	20 (100.0)	-
Esophageal dysfunction, n (%)	-	20 (52.6)	-	5 (25.0)	-
Calcinosis, n (%)	-	2 (5.26)	-	6 (30.0)	-
Synovitis, n (%)	-	10 (26.3)	-	6 (30.0)	-

RA: rheumatoid arthritis; ILD: interstitial lung disease; SSc: systemic sclerosis; IPF: idiopathic pulmonary fibrosis; SD: standard deviation; CRP: C-reactive protein; ESR: erythrocyte sedimentation rate; RF: rheumatoid factor; ACPA: anti-cyclic citrullinated peptide antibodies; ANA: anti-nuclear antibodies; ACA: anti-centromere antibodies; ATA: anti-topoisomerase I antibodies; CTD: connective tissue disease; FVC: forced vital capacity; FEV1: forced expiratory volume in one second; DLCO: diffusing capacity of the lung for carbon monoxide; HRCT: high resolution computed tomography; UIP: usual interstitial pneumonia; NSIP: non-specific interstitial pneumonia; csDMARDs: conventional synthetic disease-modifying anti-rheumatic drugs; bDMARDs: biologic disease-modifying anti-rheumatic drugs.
