# Supplementary material for: Matrix metalloproteinases and their tissue inhibitors as upcoming biomarker signatures of connective tissue diseases-related interstitial lung disease: towards an earlier and accurate diagnosis
Source: Mol Med. 2025 Feb 20;31:70. doi: 10.1186/s10020-025-01128-2 (PMC11844142; doi:10.1186/s10020-025-01128-2)
Supplement: Supplementary file 2 — Additional File 2: Table S2. ROC curves analysis for the discrimination of CTDs-ILD+ from CTDs-ILD- and CTDs-ILD+ from IPF. [file 10020_2025_1128_MOESM2_ESM.pdf]

Table S2. ROC curves analysis for the discrimination of CTDs-ILD$^+$ from CTDs-ILD$^-$ and CTDs-ILD$^+$ from IPF.

		AUC (95% CI)	p-value	Optimal cut-off value (pg/mL)	Sensitivity (%)	Specificity (%)
	**RA-ILD$^+$ vs RA-ILD$^-$**					
	MMP-2	0.7188 (0.6001-0.8374)	**0.0023**	> 169948	54.17	88.00
	MMP-7	0.8996 (0.8207-0.9785)	**<0.0001**	> 15806	87.76	84.00
	MMP-9	0.7433 (0.6291-0.8576)	**0.0007**	> 347110	56.25	84.00
	MMP-10	0.6967 (0.5718-0.8215)	**0.0061**	> 523.8	64.58	72.00
**CTDs-ILD$^+$ *vs* CTD-ILD$^-$**	MMP-12	0.7355 (0.6157-0.8553)	**0.0013**	> 146.8	60.87	79.17
	TIMP-1	0.6599 (0.5350-0.7847)	**0.0273**	< 56004	59.18	70.83
	**SSc-ILD$^+$ vs SSc-ILD$^-$**					
	MMP-7	0.8816 (0.7962-0.9669)	**< 0.0001**	> 21564	71.05	95.00
	MMP-9	0.9324 (0.8707-0.9941)	**< 0.0001**	> 302893	78.38	100.00
	MMP-10	0.7132 (0.5738-0.8525)	**0.0081**	> 382.1	81.58	55.00
	MMP-12	0.7061 (0.5711-0.8410)	**0.0108**	> 203.7	40.54	100.00
	**RA-ILD$^+$ vs IPF**					
	MMP-3	0.6504 (0.5356-0.7652)	**0.0166**	> 58249	57.10	73.70
	MMP-7	0.8059 (0.7146-0.8971)	**<0.0001**	< 35114	65.30	84.20
	MMP-9	0.7292 (0.6228-0.8356)	**0.0003**	< 414407	58.33	86.84
	MMP-10	0.6499 (0.5332-0.7666)	**0.0174**	< 567.0	43.75	84.21
**CTDs-ILD$^+$ vs IPF**	TIMP-1	0.6949 (0.5853-0.8046)	**0.0018**	< 57973	63.27	74.36
	TIMP-2	0.6223 (0.5045-0.7401)	0.0507	-	-	-
	**SSc-ILD$^+$ vs IPF**					
	MMP-1	0.5796 (0.4504-0.7088)	0.2293	-	-	-
	MMP-7	0.7462 (0.6370-0.8554)	**0.0002**	< 33983	55.30	84.20
	MMP-10	0.7071 (0.5908-0.8234)	**0.0019**	< 1007	86.84	47.37
	MMP-12	0.5836 (0.4532-0.7140)	0.2131	-	-	-

ROC: receiver operating characteristic; CTDs: connective tissue diseases; ILD: interstitial lung diseases; IPF: idiopathic pulmonary fibrosis; AUC: area under the curve; CI: confidence interval; RA: rheumatoid arthritis; SSc: systemic sclerosis; MMP: matrix metalloproteinases; TIMP: tissue inhibitors of metalloproteinases. Significant results are highlighted in **bold**.
