# Supplementary figures and images for: Matrix metalloproteinases and their tissue inhibitors as upcoming biomarker signatures of connective tissue diseases-related interstitial lung disease: towards an earlier and accurate diagnosis
Source: Mol Med. 2025 Feb 20;31:70. doi: 10.1186/s10020-025-01128-2 (PMC11844142; doi:10.1186/s10020-025-01128-2)

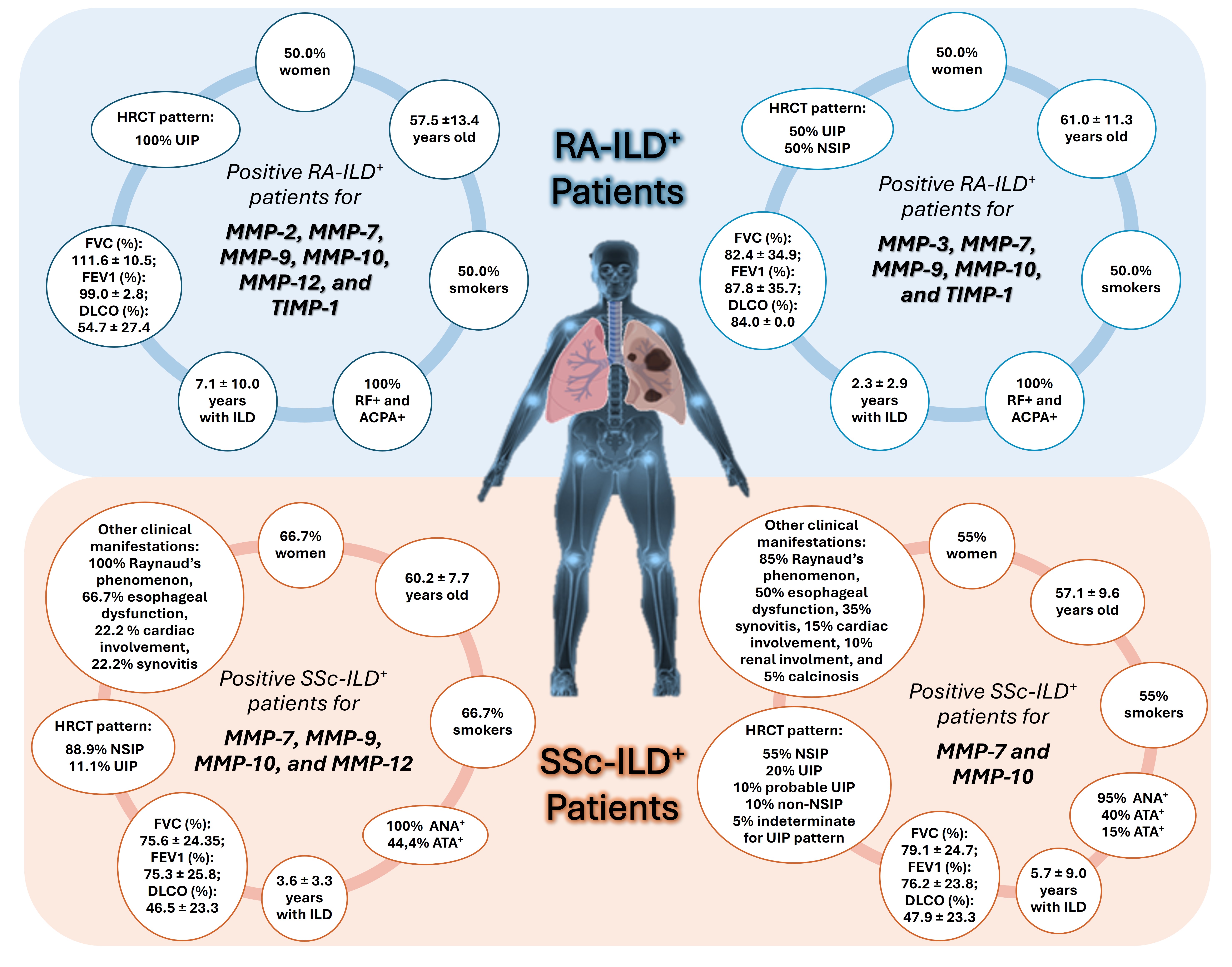

Supplement: Supplementary file 3 — Additional File 3: Figure S1. Clinical characteristics of patients with RA-ILD+ and SSc-ILD+ who are combinatorial-marker-positive for their characterization versus patients with RA-ILD- and SSc-ILD-, respectively, and idiopathic pulmonary fibrosis patients. RA: rheumatoid arthritis; ILD: interstitial lung disease; HRCT: high resolution computed tomography; UIP: usual interstitial pneumonia; NSIP: non-specific interstitial pneumonia; FVC: forced vital capacity; FEV1: forced expiratory volume in one second; DLCO: diffusing capacity of the lung for carbon monoxide; RF: rheumatoid factor; ACPA: anti-cyclic citrullinated peptide antibodies; SSc: systemic sclerosis; ANA: anti-nuclear antibodies; ACA: anti-centromere antibodies; ATA: anti-topoisomerase I antibodies. [file 10020_2025_1128_MOESM3_ESM.jpg]

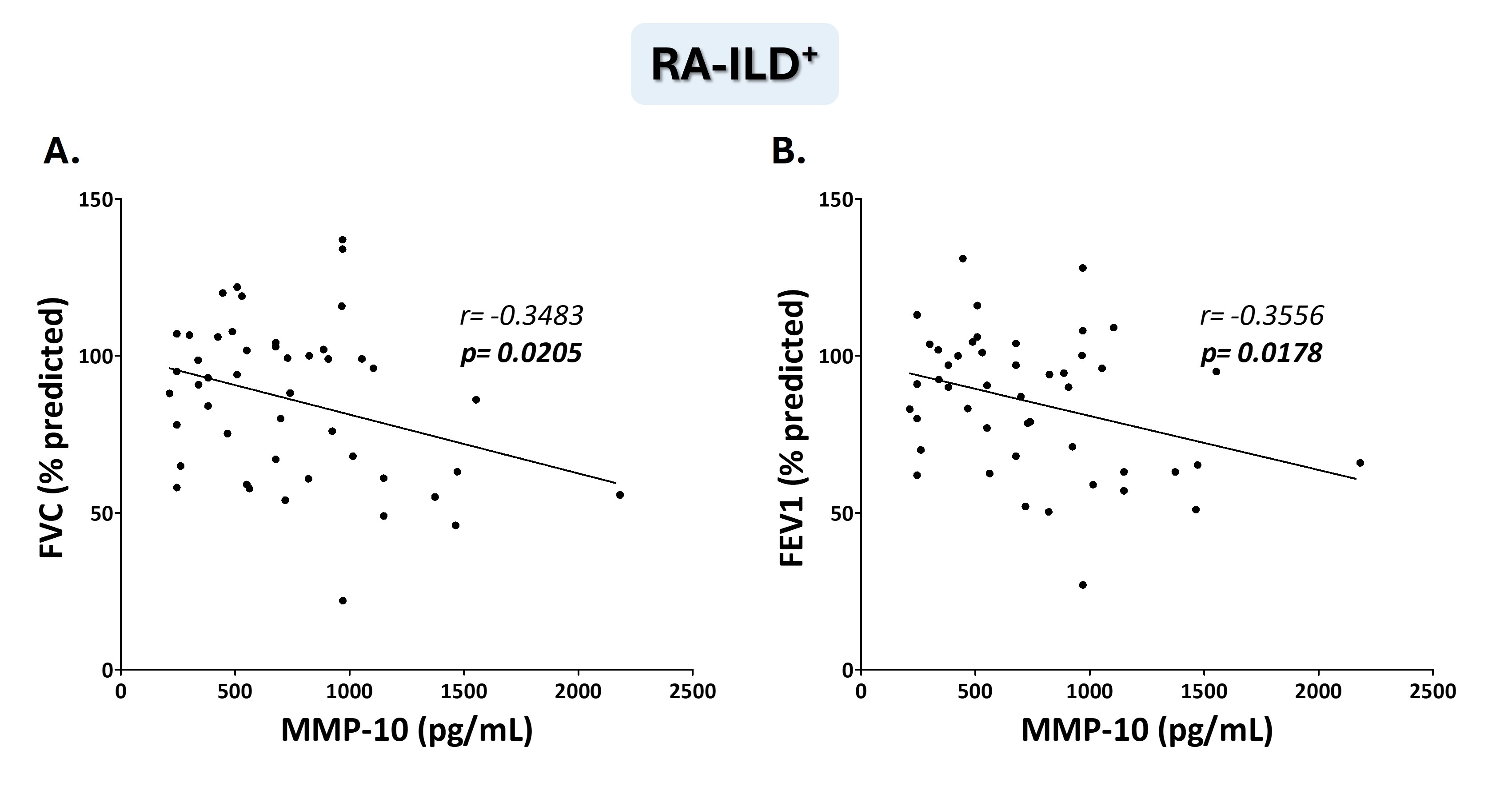

Supplement: Supplementary file 4 — Additional File 4: Figure S2. Relationship of MMP-10 serum levels with FVC (A) and FEV1 (B) in patients with RA-ILD+. MMP: matrix metalloproteinase; FVC: forced vital capacity; FEV1: forced expiratory volume in one second; RA: rheumatoid arthritis; ILD: interstitial lung disease. Significant results are highlighted. [file 10020_2025_1128_MOESM4_ESM.jpg]

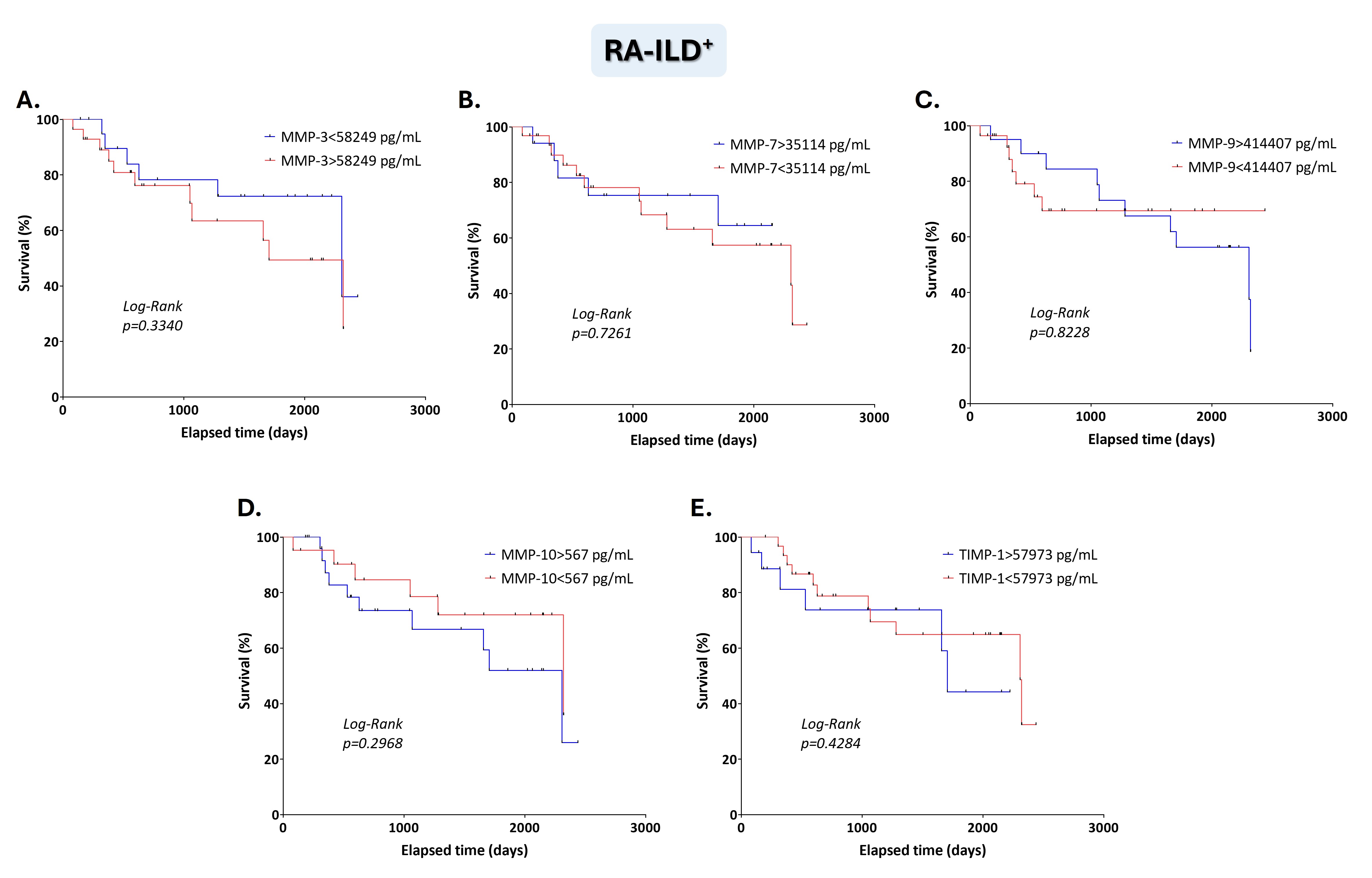

Supplement: Supplementary file 5 — Additional File 5: Figure S3. Survival (time to death or lung transplantation) according to the serum level* of MMP-3 (A), MMP-1 (B), MMP-9 (C), MMP-10 (D), and TIMP-1 (E) in patients with RA-ILD+. RA: rheumatoid arthritis; ILD: interstitial lung disease; MMPs: matrix metalloproteinases; TIMPs: Matrix metalloproteinases inhibitors. *The cut-off of MMPs and TIMP levels was determined by ROC curves for discriminating RA-ILD+ and idiopathic pulmonary fibrosis. [file 10020_2025_1128_MOESM5_ESM.jpg]

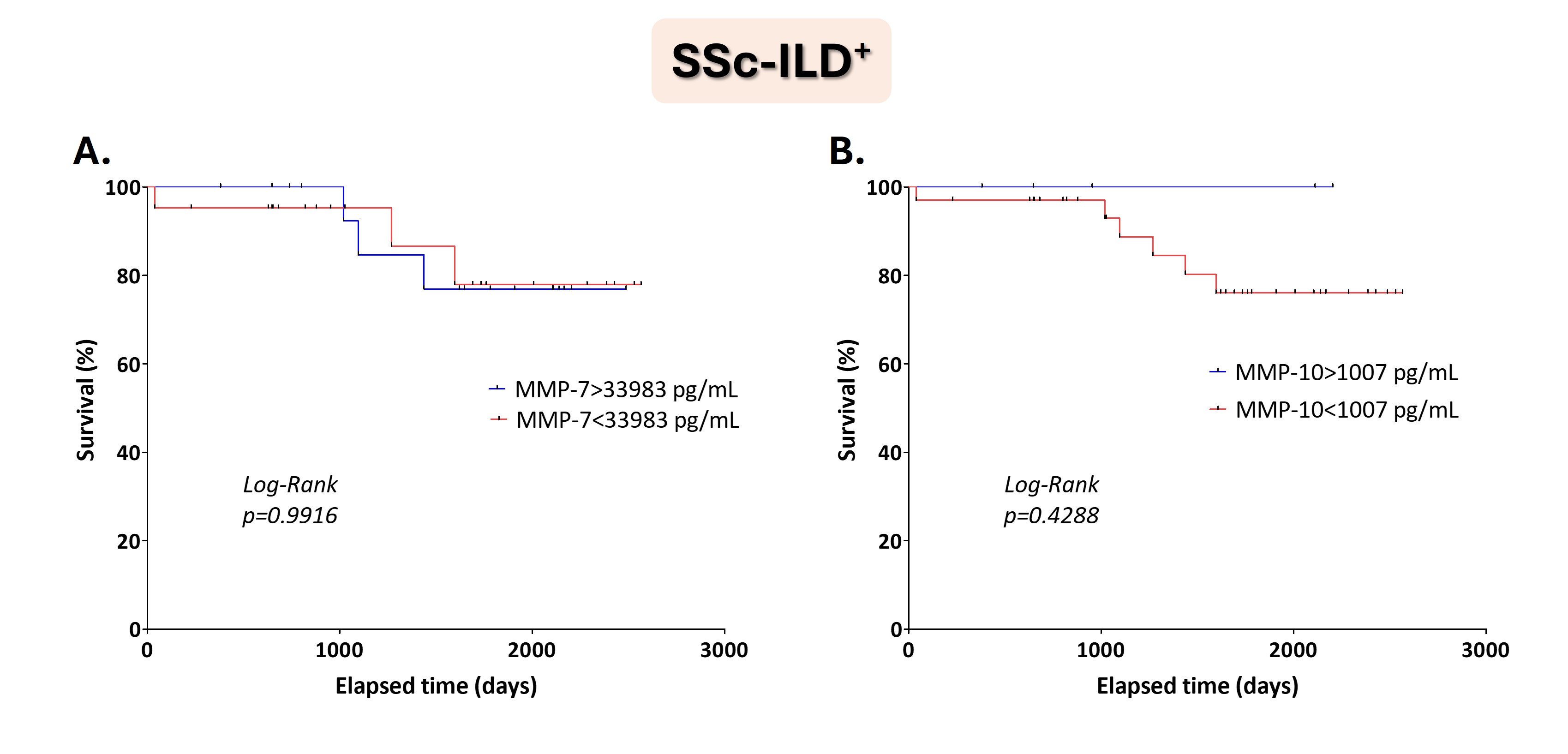

Supplement: Supplementary file 6 — Additional File 6: Figure S4. Survival (time to death or lung transplantation) according to the serum level* of MMP-7 (A) and MMP-10 (B) in patients with SSc-ILD+. SSc: systemic sclerosis; ILD: interstitial lung disease; MMPs: matrix metalloproteinases; TIMPs: Matrix metalloproteinases inhibitors. *The cut-off of MMP levels was determined by receiver operating characteristic curves for discriminating SSc-ILD+ and idiopathic pulmonary fibrosis. [file 10020_2025_1128_MOESM6_ESM.jpg]
